# A Dynamic Core in Human NQO1 Controls the Functional and Stability Effects of Ligand Binding and Their Communication across the Enzyme Dimer

**DOI:** 10.3390/biom9110728

**Published:** 2019-11-12

**Authors:** Pavla Vankova, Eduardo Salido, David J. Timson, Petr Man, Angel L. Pey

**Affiliations:** 1 Institute of Microbiology, Academy of Sciences of the Czech Republic, Videnska 1083, 142 20 Prague 4, Czech Republic; pavla.vankova@biomed.cas.cz; 2 Department of Biochemistry, Faculty of Science, Charles University, Hlavova 2030/8, 128 43 Prague 2, Czech Republic; 3 Center for Rare Diseases (CIBERER), Hospital Universitario de Canarias, Universidad de La Laguna, 38320 Tenerife, Spain; edsalido@gmail.com; 4 School of Pharmacy and Biomolecular Sciences, University of Brighton, Huxley Building, Lewes Road, Brighton BN2 4GJ, UK; D.Timson@brighton.ac.uk; 5 Department of Physical Chemistry and Unit of Excellence in Chemistry, University of Granada, Av. Fuentenueva s/n, E-18071 Granada, Spain

**Keywords:** protein structural dynamics, NQO1, ligand binding, protein stability, allostery, protein degradation

## Abstract

Human NAD(P)H:quinone oxidoreductase 1 (NQO1) is a multi-functional protein whose alteration is associated with cancer, Parkinson’s and Alzheimer´s diseases. NQO1 displays a remarkable functional chemistry, capable of binding different functional ligands that modulate its activity, stability and interaction with proteins and nucleic acids. Our understanding of this functional chemistry is limited by the difficulty of obtaining structural and dynamic information on many of these states. Herein, we have used hydrogen/deuterium exchange monitored by mass spectrometry (HDXMS) to investigate the structural dynamics of NQO1 in three ligation states: without ligands (NQO1_apo_), with FAD (NQO1_holo_) and with FAD and the inhibitor dicoumarol (NQO1_dic_). We show that NQO1_apo_ has a minimally stable folded core holding the protein dimer, with FAD and dicoumarol binding sites populating binding non-competent conformations. Binding of FAD significantly decreases protein dynamics and stabilizes the FAD and dicoumarol binding sites as well as the monomer:monomer interface. Dicoumarol binding further stabilizes all three functional sites, a result not previously anticipated by available crystallographic models. Our work provides an experimental perspective into the communication of stability effects through the NQO1 dimer, which is valuable for understanding at the molecular level the effects of disease-associated variants, post-translational modifications and ligand binding cooperativity in NQO1.

## 1. Introduction

Human NAD(P)H:quinone oxidoreductase 1 (NQO1; EC 1.6.5.2) is a multifunctional stress protein mostly localized in the cellular cytosol [1]. NQO1 expression is upregulated as a response to different types of cellular stress and through several mechanisms, including the antioxidant response through Nrf2-mediated and Ah2 signaling pathways [1,2,3,4].

**Figure 1:**
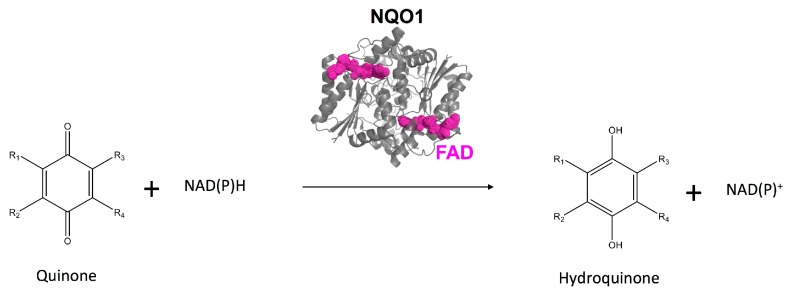
NQO1 catalyzes the two-electron reduction of a wide range of quinones. This enzyme also reduces other organic and inorganic species, such as superoxide radicals and iron(III) ions.

NQO1 displays multiple enzymatic and non-enzymatic functions [1,2,3,4,5]. NQO1 catalyzes different reactions with cytoprotective and metabolic roles, such as the two-electron reduction of quinones to form hydroquinones [2,6] (Figure 1), redox cycling of quinones [2], reduction of coenzyme Q_10_ and vitamin E to their antioxidant form [2], scavenging reactive oxygen species [1,2,3,7], reduction of catecholamines and vitamin K [1,2] and maintenance of the NADH/NAD^+^ redox balance [3,8]. The main features of these biochemical reactions involving NQO1 have been investigated in detail mainly through enzymological and structural analyses. Structurally, the enzyme forms obligate functional homodimers, with two active sites located in the monomer:monomer interface (MMI), and each monomer consists of two different domains: (i) an N-terminal domain spanning residues 1–224 that contains part of the active site and is involved in the tight binding of one FAD molecule per monomer and protein dimerization; (ii) a C-terminal domain (residues 225–274) that contributes to stabilizing the protein dimer and to the binding of the NAD(P)H coenzyme and the substrates [1,4,9,10,11,12,13,14]. The functional cycle of NQO1 generally involves two steps according to a *ping-pong* mechanism: first, in the reductive half-reaction, an NAD(P)H molecule binds to the enzyme and rapidly reduces the FAD to FADH_2_ (with a second-order rate constant of ~10^6^ M^−1^·s^−1^) with the subsequent release of the oxidized nicotinamide dinucleotide; and second, in a very fast oxidative half-reaction (with a second-order rate constant >10^9^ M^−1^·s^−1^), the substrate binds and is reduced by the FADH_2_, thus regenerating the flavin in oxidized form and releasing the reduced product [1,10]. This catalytic cycle is known to be inhibited by different coumarin-based molecules (the best characterized is the biscoumarin, dicoumarol) that act as competitive inhibitors by blocking the NAD(P)H access to the active site and partially occupying the NAD(P)H binding site [1,15]. Importantly, comparison of the crystal structures of NQO1 with FAD bound (NQO1_holo_) with that containing also dicoumarol bound (NQO1_dic_) have revealed that inhibitor binding causes only minor structural rearrangements in the conformation, which localize at the surface of the catalytic site [15]. Among non-enzymatic functions, we must highlight the ability of NQO1 to develop protein: protein and protein: RNA interactions [1,2,16,17,18,19,20]. In particular, protein:protein interactions involving NQO1 are relevant to understand its multiple roles in physiological and pathological processes. NQO1 interacts with key transcription factors associated with cancer (e.g., p53, p73α and HIF-1α) [17,18] and proteins involved in HIV infection (e.g., Tat protein) [19], and these interactions increase the intracellular stability of these protein partners by preventing their degradation by the proteasome. These protein:protein interactions presumably depend on the functional state of NQO1: binding of NADH may increase the strength of these interactions, while dicoumarol binding has the opposite effect [9,17,19]. In addition, NQO1_holo_ binds to the 20S particle of the proteasome and inhibits its proteolytic activity, while FAD withdrawal (i.e., NQO1_apo_) renders NQO1 susceptible to degradation by this mechanism [16].

Alterations in NQO1 stability and function are associated to different extents with a variety of human diseases, including cancer, neurological disorders (such as Parkinson´s and Alzheimer´s diseases, multiple sclerosis and schizophrenia) and cardiovascular diseases [1,21]. In these cases, either the wild-type (WT) NQO1 protein and/or a common polymorphic variant (rs1800566, causing a Pro187Ser amino acid exchange) have been found to be associated with increased disease predisposition. The Pro187Ser variant decreases NQO1 activity due to a large defect in FAD binding (10- to 40-fold lower affinity than that of WT) and in conformational stability, leading to its rapid intracellular degradation by the proteasome [1,10,14,16,22,23,24,25]. In general, reduced NQO1 activity or protein levels are commonly observed under these pathological conditions [1,26], although for the particular case of cancer, overexpression of NQO1 is also associated with cancer progression, which makes pharmacological inhibition of NQO1 (e.g., by dicoumarol or related compounds) a potential therapeutic strategy to treat this disease if it selectively targets cancer cells [27,28,29]. Linked to some of these pathological conditions, the intracellular stability of NQO1 WT is controlled by the population of the NQO1_apo_ state, which is efficiently targeted to the ubiquitin-dependent proteasomal degradation pathway [14,25,30]. Recent works also demonstrated that alterations in the phosphorylation pattern of NQO1 WT at different sites might be associated with these pathological states, likely through effects on the FAD binding affinity and consequently on the intracellular stability of NQO1 WT [26,30,31].

NQO1 is an excellent model to decipher the role of protein dynamics in the function and stability of flavin-dependent enzymes, the role of ligand binding in disease-associated protein stability, and the molecular mechanisms by which mutations cause loss-of-function genetic diseases [1,5,9,10,14,25,30,31,32,33,34]. FAD binding to NQO1 WT triggers a large conformational change, which can be observed by some biophysical techniques (circular dichroism, infrared and NMR spectroscopies or small-angle X-ray scattering), and increases the kinetic stability of the protein dimer, although high-resolution structural information is only available for the NQO1_holo_ state [9,10,11,14,22,34,35]. This structural change is accompanied by significant changes in overall protein flexibility (evidence provided by proteolysis experiments and structure-based analyses of FAD binding energetics) [10,14,34], presumably linked to the fast degradation of NQO1_apo_ vs. NQO1_holo_ in the cell [14,25], although no high-resolution experimental information on these dynamic changes is available [14]. Regarding dicoumarol binding, the comparison of the X-ray crystallographic structures of NQO1_holo_ and NQO1_dic_ has revealed only local changes in protein structure at the active site [15], and thus, this did not provide details on the remarkable stabilizing effect of dicoumarol binding on the overall conformational stability and the dynamics of the C-terminal domain [14]. A critical role of protein dynamics in the mechanisms causing alterations in NQO1 function due to the Pro187Ser polymorphism and other rare cancer-associated mutations, phosphorylation at specific sites as well as the effect of suppressor mutations of the Pro187Ser phenotype have been put forward from experimental and computational studies [5,9,10,14,30,31,32,33,34,36]. Thus, they also await high-resolution information on the changes in protein dynamics due to these site-specific changes in different ligation states (NQO1_apo_, NQO1_holo_ and NQO1_dic_).

We report herein a detailed experimental analysis on the structural dynamics of human NQO1 in three functionally relevant ligation states (NQO1_apo_, NQO1_holo_ and NQO1_dic_). Our results uncover the existence of a dynamic network within the NQO1 dimer that readily responds to the binding of functional ligands and help to explain their effects on NQO1 function and stability *in vitro* and *in vivo*. Our work also provides an experimental benchmark to understand the allosteric effects of disease-associated variants, post-translational modifications and ligand binding in NQO1.

## 2. Materials and Methods

### 2.1. Protein Expression and Purification

Protein expression and purification was carried out as described [30]. *E. coli* BL21(DE3) cells were transformed with pET46 Ek/LIC vector containing the cDNA of human WT NQO1 [22] and grown for 16 h in LBA medium (LB containing 0.1 mg·ml^−1^ ampicillin at 37 °C). This culture was diluted 40-fold in fresh LBA and grown at 37 °C for 3 h. Expression was then triggered by the addition of IPTG (isopropyl β-D-1-thiogalactopyranoside) at a final concentration of 0.5 mM. Induced cells were incubated for 6 h at 25 °C, harvested by centrifugation, washed with binding buffer (BB, 20 mM sodium phosphate, 300 mM NaCl and 50 mM imidazole at pH 7.4) and frozen overnight at −80 °C. Then, cells were thawed and resuspended in BB containing 1 mM PMSF (phenylmethylsulfonyl fluoride) and lysed by sonication. Crude extracts were clarified by centrifugation (20 min at 20,000× g and 4 °C), and supernatants were loaded into immobilized metal affinity chromatography (IMAC) columns (GE Healthcare) equilibrated in BB. Columns were washed with BB, and the protein was eluted with elution buffer (BB containing 500 mM imidazole). The eluate was exchanged to 50 mM K-HEPES (2-[4-(2-hydroxyethyl)piperazin-1-yl]ethanesulfonic acid, potassium salt) pH 7.4 using PD-10 columns (GE Healthcare), centrifuged for 30 min at 20,000× *g* and 4 °C, and the UV–visible spectra of the supernatants were registered in a HP8453 UV–Visible spectrophotomer (Agilent). This purified holo-protein containing high levels of FAD bound (70%–80% based on the absorbance ratio at 450 nm and 280 nm; see also [30,33,36]) was stored at −80 °C upon flash freezing in liquid N_2_. Further purification of the NQO1 dimer was carried out by size-exclusion chromatography using a HiLoad^®^ 16/600 Superdex^®^ 200 prep grade (GE Healthcare) and using 20 mM K-HEPES 200 mM NaCl at pH 7.4 as the mobile phase. This purified protein was subsequently used to obtain apo-protein upon treatment with BB containing 2 M urea and 2 M KBr, 1 mM DTT (1,4-dithiothreitol) and 1 mM PMSF at 4 °C, and separation of the apo-protein and the FAD released was carried out by IMAC at 4 °C. Apo-proteins were finally exchanged to 50 mM K-HEPES at pH 7.4 using PD-10 columns at 4 °C, concentrated using VIVASPIN 6 30,000 MWCO PES devices (Sartorius) and stored at −80 °C after flash freezing in liquid N_2_. The purity and integrity of the holo- and apo-protein was checked by polyacrylamide gel electrophoresis in the presence of sodium dodecylsulphate (SDS-PAGE; Figure S1). The holo-protein was also found to display enzyme kinetic parameters (Figure S1) consistent with those previously reported using the same protein construct and purification procedure [9,22].

Purified proteins were also verified by mass spectrometry. The intact mass was analyzed through direct infusion on ESI-FT-ICR MS (Figure S2), showing that the protein was expressed intact and lacking the N-terminal Met. The dimeric state of NQO1_apo_ and NQO1_holo_ was verified by native ESI-MS (Figure S3).

### 2.2. Hydrogen/Deuterium Exchange Mass Spectrometry (HDXMS)

Amide hydrogen/deuterium exchange (HDX) of NQO1 was followed for its apo (NQO1_apo_) and holo (NQO1_holo_) forms, and the holo form was also analyzed in the presence of dicoumarol (NQO1_dic_). Prior to the exchange, the NQO1_holo_ and NQO1_dic_ at 20 μM concentration were pre-incubated with 10 molar excess of FAD for 5 min. NQO1_dic_ was then further mixed with 10 molar excess of dicoumarol and incubated for another 5 min. The exchange reaction was initiated by a 10-fold dilution into a D_2_O-based 50 mM K-HEPES, pD 7.4, 1 mM TCEP (tris(2-carboxyethyl)phosphine). The exchange was thus followed at 2 μM protein concentration. Deuterium labelling was quenched by 0.5 M Glycine-HCl, pH 2.3, which was added at 1:1 ratio. The samples were then frozen in liquid N_2_. Exchange was followed for 10 s, 30 s, 2 min, 5 min, 20 min, 1 h and 3 h, where 10 s, 5 min and 3 h samples were done in replicate. Each sample was quickly thawed and injected into a cooled LC system. Here, the protein was digested on custom-made nepenthesin-2 (Nep-2) and pepsin columns coupled in tandem (each having a bed volume of 66 μL), and the resulting peptides were trapped on a VanGuard Pre-column (ACQUITY UPLC BEH C18, 130Å, 1.7 µm, 2.1 mm × 5 mm, Waters, Milford, MA, USA), where they were desalted. The solvent used for digestion and desalting (0.4% formic acid (FA) in water) was pumped by a 1260 Infinity II Quaternary pump (Agilent Technologies, Waldbronn, Germany) at a flow rate of 200 μL·min^−1^. After three minutes of digestion and desalting, the peptides were separated on an analytical column (ACQUITY UPLC BEH C18, 130 Å, 1.7 µm, 1 mm × 100 mm, Waters, Milford, MA, USA) using linear gradient (5%–45% B in 7 min) followed by a quick step to 99% B lasting 5 min. Solvent A was 0.1% FA/2% acetonitrile (ACN) in water, B was 0.1% FA/98% ACN in water. The gradient was delivered by the 1290 Infinity II LC System (Agilent Technologies, Waldbronn, Germany) at a flow of 40 μL·min^−1^. Digestion, desalting and separation were done at 0 °C and pH 2.3 to minimize deuterium loss. The LC system was connected directly to an electrospray ionization source of a 15T FT-ICR mass spectrometer (solarix XR, Bruker Daltonics, Bremen, Germany) operating in broad-band MS mode. Data were peak-picked and exported using DataAnalysis v. 5.0 (Bruker Daltonics, Bremen, Germany) and then processed by the in-house developed program Deutex (unpublished). Peptides arising from the digestion were identified through separate data-dependent LC-MS/MS analyses (using the same setup as described above) and database searching by the MASCOT algorithm v. 2.4 (Matrix Science, London, United Kingdom) against a custom-build database containing sequences of pepsin, nepenthesin-2 and NQO1. Fully deuterated samples were prepared and used for back-exchange correction as described previously [37,38]. Differences in deuteration can be considered as significant if they exceed 0.25 Da (calculated as 3× the average standard deviation).

**Figure 2:**
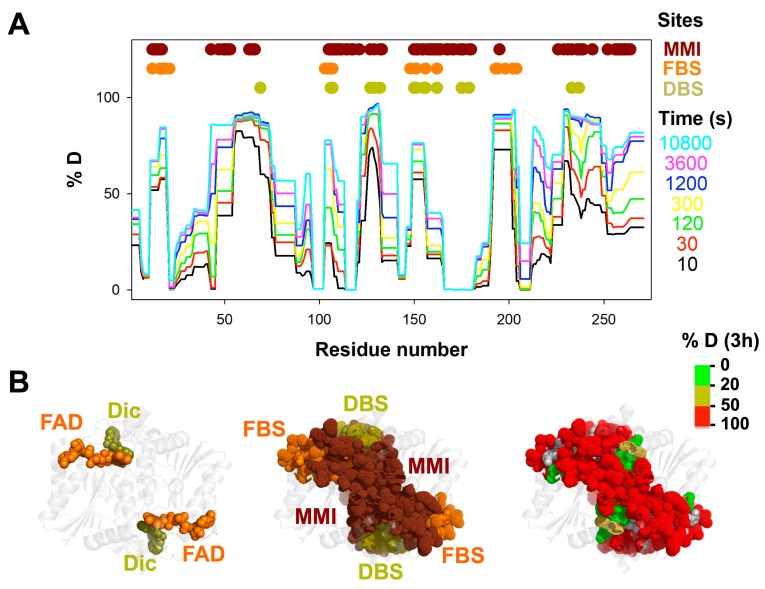
Overall HDX kinetics of NQO1_apo_. (**A**) HDX kinetics for segments of NQO1_apo_. Residues belonging to the monomer: monomer interface (MMI), FAD binding site (FBS) and dicoumarol binding site (DBS) are indicated as colored circles. These sites were retrieved from the analysis of the NQO1 structure (PDB 2F1O; [15]) using the PISA server (https://www.ebi.ac.uk/pdbe/pisa/); (**B**) Structural representation of HDX after 3 h [%D (3 h)] in NQO1_apo_. For visual aid, the left panel shows the location of bound FAD and dicoumarol, the middle panel shows those residues belonging to the MMI, FBS and DBS, and the right panel displays the %D after 3 h for residues belonging to these functional sites.

**Figure 3:**
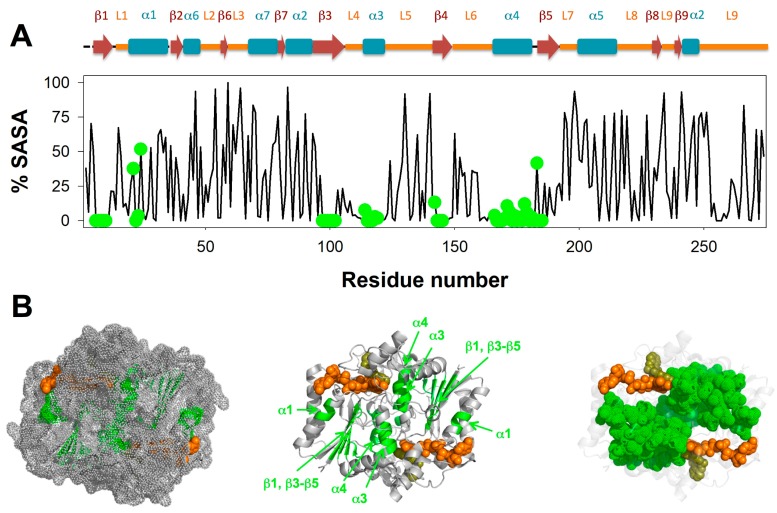
Non-exchanging segments define a minimally stable core in NQO1_apo_. (**A**) Plot of the % SASA (solvent accessible surface area) for individual residues (considering backbone and side-chain) calculated from the structure of NQO1_dic_ (PDB 2F1O [15]) using GETAREA (http://curie.utmb.edu/getarea.html; this algorithm does not consider the ligands in the calculation). Secondary structure elements are depicted according to [11]; residues belonging to non-exchanging segments are displayed as green circles; (**B**) Structural representation of non-exchanging segments (using PDB 2F1O [15]). The left panel shows a surface representation highlighting the burial of the minimally stable core. The middle panel shows segments belonging to this core plotted onto secondary structure elements. The right panel shows that this core may contribute to the stable folding of the individual monomers as well as their assembly into the dimer, with only a few stable contacts with the FAD (in orange ball representation) and the dicoumarol (in yellow ball representation).

**Figure 4:**
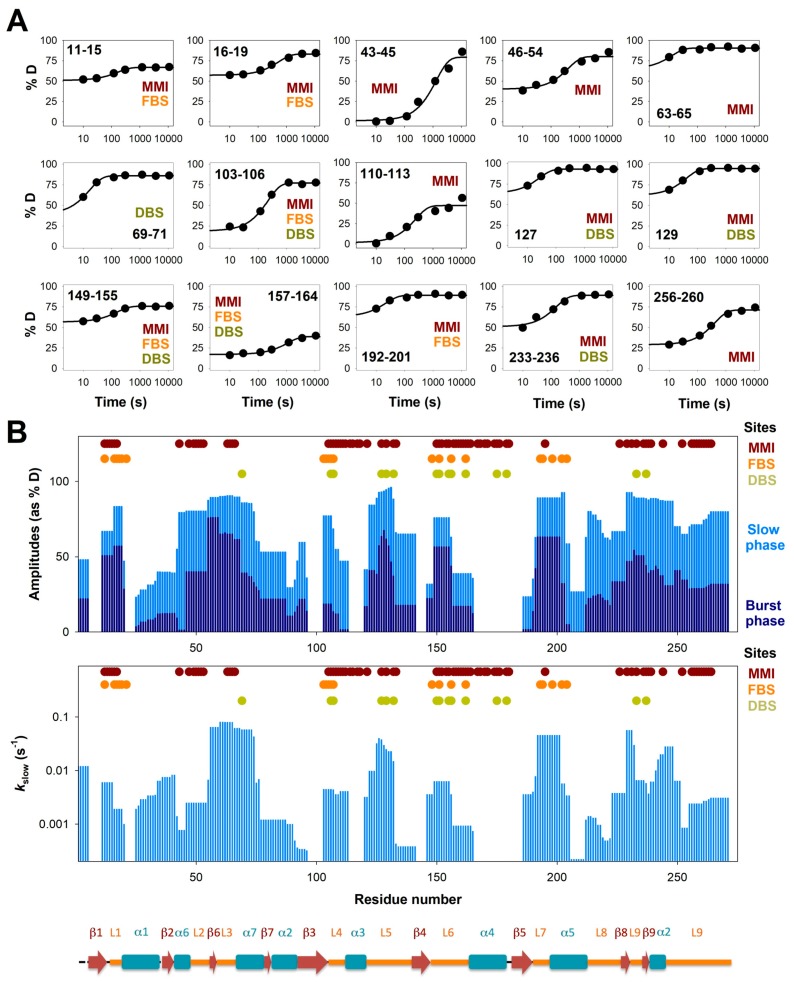
Segment-specific HDX kinetics of NQO1_apo_. (**A**) Fittings of HDX kinetics for selected segments typically showing at least 50%D incorporation after 3 h and considered part of the functional sites (MMI, FBS and DBS). (**B**) Plots of the amplitudes for the burst- and slow-phase in HDX for segments (upper panel) and rate constants for the slow phase (lower panel) for segments with at least 20%D after 3 h. The elements of the secondary structure along the protein sequence are also indicated.

**Figure 5:**
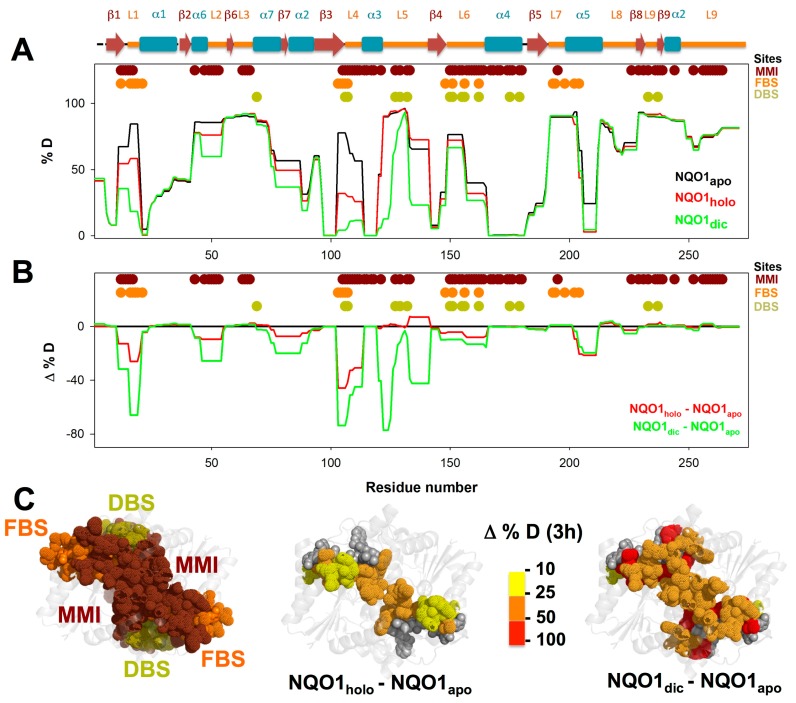
Overall HDX kinetics for segments of NQO1 upon binding FAD and dicoumarol. (**A**,**B**) HDX for segments of NQO1_apo_, NQO1_holo_ and NQO1_dic_. Panel A shows %D after 3 h, and Panel B the difference in this parameter (Δ%D) between NQO1_holo_ or NQO1_dic_ and NQO1_apo_. Residues belonging to the MMI, FBS and DBS are indicated (retrieved as described in Figure 2). (**C**) Δ%D after 3 h in NQO1 upon FAD and dicoumarol binding plotted onto the NQO1 structure (PDB 2F1O). For comparison, the left panel shows the location of MMI, FBS and DBS, the middle and right panels show those residues belonging to the sites that displayed at least a decrease of 10%D after 3 h in NQO1_holo_ (middle panel) or NQO1_dic_ (right panel) vs. NQO1_apo_.

**Figure 6:**
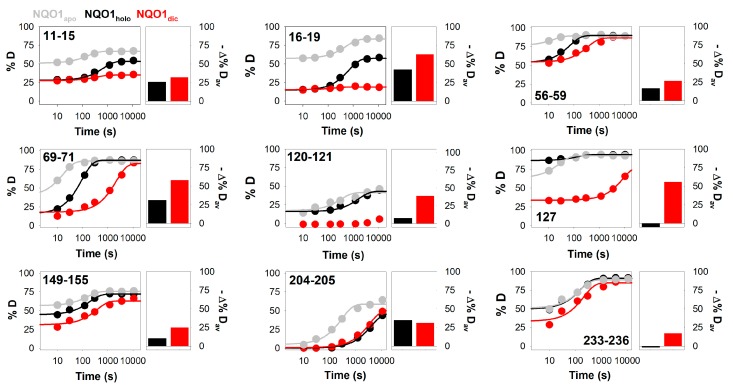
Changes in segment-specific HDX kinetics of NQO1 upon FAD and dicoumarol binding. Right panels show fittings of HDX kinetics for selected individual segments using a single exponential function with a burst-phase for NQO1_apo_ (**grey**), NQO1_holo_ (**black**) and NQO1_dic_ (**red**). These data were used to calculate Δ%D_av_, as an average of the time points with maximal differences between a given NQO1 form (NQO1_holo_, black; NQO1_dic_, red) and NQO1_apo_, as a single metric to quantify changes in HDX kinetics (left panels).

**Figure 7:**
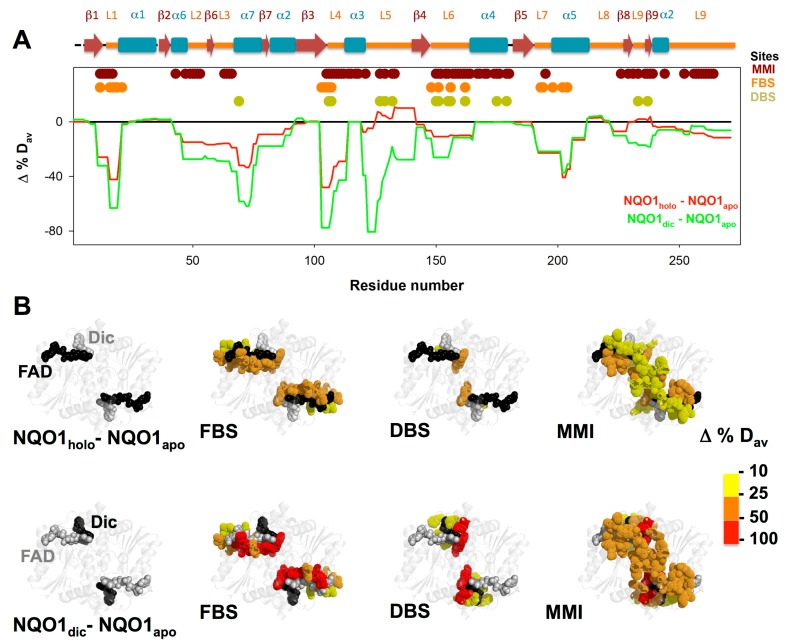
Changes in HDX kinetics of NQO1 upon binding FAD and dicoumarol as changes in %D_av_ (Δ%D_av_). (**A**) Δ%D_av_ for NQO1 segments upon binding FAD (NQO1_holo_) and dicoumarol (NQO1_dic_), using NQO1_apo_ as a reference. Residues belonging to the MMI, FBS and DBS are indicated (retrieved as in Figure 2). (**B**) Representation of Δ%D_av_ onto the structure of NQO1 (using PDB 2F1O). The upper row shows results for the Δ%D_av_ of NQO1_holo_ and the lower row represents NQO1_dic_. Different panels in each row show results for residues involved in the FBS, DBS or MMI.

The optimization of digestion conditions, including numerous proteolytical setups, showed that the serial combination of nepenthesin-2 with pepsin, operated at 200 µL·min^−1^, provided the best results in terms of sequence coverage (98.9%, missing the last three amino acids), number of peptides (140), average peptide length (8.3) and redundancy (4.1) (Figure S4). At this point, it should be also noted that the region between 100 and 110 yielded peptides of considerable hydrophobicity for which the signal intensity/quality was just at the threshold level, and thus conclusions derived from their analyses must be made with caution. The redundant peptide set was used to calculate deuteration in the shortest possible segments using the overlapping peptides. This analysis provided more detailed non-redundant information (high-resolution set). Data in this high-resolution set are mainly described in the manuscript (Figure 2, Figure 3, Figure 4, Figure 5, Figure 6 and Figure 7, Figure S6 and Table S1), while those using experimental peptides (low resolution set) are found in Figures S7–S11 and Table S2. Note that the high-resolution set essentially leads to the same key conclusions as the low resolution one but, in principle, the former narrows the region for which HDX kinetics is assessed.

To report data on NQO1 segments from HDXMS, we did not consider the His-tag and used the native sequence from Met1 to Lys274. Therefore, the numbering used throughout the manuscript differs from that reported in some crystal structures of NQO1, which did not include Met1 and thus numbered the residues from Val1 to Lys273 (Val2 to Lys274 in the native sequence).

## 3. Results and Discussion 

### 3.1. A Stable Folded Core in NQO1_apo_ with Highly Dynamic Functional Sites

**Scheme 1:**
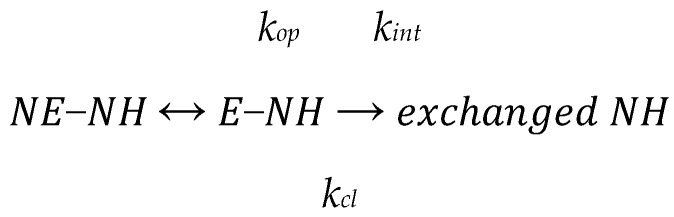
Linderstrøm-Lang model for HDX kinetics.

The results of HDX kinetics for NQO1_apo_ are shown in Figure 2A. Results are presented as the % of the maximal (theoretical) deuterium incorporation for each segment (%D). Virtually all peptides characterized in this work for NQO1 complied with EX2 behavior (the presence of a tiny contribution from the EX1 regime can be detected in a few NQO1 peptides; see Figure S5). In the EX2 mechanism, the intrinsic exchange rate constant (k_int_) is much lower than the rate constant (k_cl_) for the conversion between non-exchanging (NE-NH) and exchanging (E-NH) states, according to the Linderstrøm-Lang model (Scheme 1):

Assuming a pure EX2 behavior, the rate constant for exchange of individual backbone amides would be equal to the product of the equilibrium constant between NE-NH and E-NH (K_op_ = k_op_/k_cl_) and k_int_ [39]. Therefore, for this very simple mechanism (note that the conformational equilibrium is simply two-state and experimental HDX is rarely pure EX2), the experimental rate constant for exchange reflects to some extent the local stability (e.g., due to hydrogen bonding and burial in the structure) of the secondary structure.

Overall, the HDX kinetics was very heterogeneous among different protein segments of NQO1_apo_ (Figure 2A). Most of the segments showed fast HDX kinetics (typically exchanging more than 20%D in the seconds-minutes time scales) while only a few peptides showed essentially no exchange after 3 h (%D < 20). Thus, in a first approach, we simply discerned between exchanging and non-exchanging segments considering the %D after 3 h (%D < 20 vs. %D ≥ 20, respectively). The functional implications of this simple analysis were considered regarding those residues in different segments belonging to three functional sites: the FAD binding site (FBS), the dicoumarol binding site (DBS) and the monomer: monomer interface (MMI)(see Figure 2A), as provided by analysis of an X-ray crystallographic structure (PDB 2F1O [15]). It is worth noting that in this structure, the FBS and DBS are located adjacent to each other in the NQO1 monomer (actually, FAD is structurally part of the DBS), and both sites are close to the MMI (Figure 2B). Importantly, most of the residues that belong to the FBS, DBS and MMI are classified as exchanging in NQO1_apo_ (%D ≥ 20; Figure 2A,B).

Within non-exchanging segments, we found that these contained mostly residues buried in the crystallographic structure of NQO1 (in a ternary complex with FAD and dicoumarol bound, PDB 2F1O; NQO1_dic_) (Figure 3A). Thus, these sequences likely represent regions that are critical for the acquisition and maintenance of a minimally stable dimeric fold in NQO1_apo_ (note that NQO1_apo_ is dimeric in solution but more expanded and flexible, and with lower conformational stability, than NQO1_holo_ [9,14]). This minimal core involves helices α1, α3 and α4 and sheets β1 and β3–β5 (Figure 3A and Figure S8). This core may also contribute to the acquisition of a minimally folded monomeric state that becomes stabilized in the dimeric state by the interactions between helices α3 and α4 across the monomers (i.e., the MMI) (Figure 3B and Figure S8).

Importantly, the FBS and DBS in NQO1_apo_ are overall exchanging (Figure 2B, Figure 3B and Figure S8B), with the main exceptions being some marginal contacts in helices α1 and α3 (FBS) and α4 (DBS) (Figure 3B and Figure S8B). Thus, our HDX analyses support that the conformational ensemble of NQO1_apo_ is essentially populated by states non-competent for FAD or dicoumarol binding due to the high structural dynamics of their binding sites [9,14,34].

### 3.2. Complex HDX Kinetics

To provide deeper insight into the structural dynamics of NQO1_apo_, we carefully analyzed the HDX kinetics for all protein segments (Figure S6). It should be noted that these HDX kinetics were very consistent with those obtained directly from peptides experimentally characterized (Figures S7 and S11). For the majority of the cases, HDX kinetics was described very well by a simple function with two kinetic phases (see Figures S6 and S7 for fittings, and Tables S1 and S2 for the best-fit values): a burst-phase corresponding to HDX mostly occurring within the experimental dead time (i.e., very few seconds), and thus characterized by a single parameter: its amplitude A_burst_; and a slow phase that occurred typically in a scale of several seconds to minutes, characterized by two parameters: its amplitude A_slow_ and an apparent first-order rate constant (k_slow_), following this equation:

$$\%\;D(t)=\;A_{burst}+A_{slow}\cdot (1-exp^{-k_{slow}\cdot t)}$$

We chose to use this phenomenological description of HDX kinetics mainly for two reasons. First, it provided a simple scenario from which, using three characteristic parameters (A_burst_, A_slow_ and k_slow_), we could compare the HDX kinetics of different segments of NQO1_apo_ (see Figure 4; note that this approach also worked very well with the HDX kinetics of NQO1_holo_ and NQO1_dic_; see Figures S6 and S7 and Tables S1 and S2). Second, although HDX kinetics analyzed using more complex functions (e.g., the two kinetic phases each containing a characteristic rate constant) may provide in some cases better fits, this would put the analyses at two intertwined risks: increasing the fitting parameters would make comparisons between behaviors more difficult, and importantly, in many cases these fittings show evident signs of overparametrization.

Kinetic analyses of HDX for protein segments considered as exchanging (>20%D after 3 h) revealed certain interesting behaviors. First, for many protein segments (of different lengths), we observed a significant contribution to the HDX kinetics from both the burst and slow phases (Figure 4 and Figure S9, and Tables S1 and S2). As indicated above, the HDX kinetics of NQO1 in all three ligation states is vastly consistent with EX2 kinetics, and thus, the observed kinetics depends to some extent on the equilibrium constant between non-exchanging and exchanging states [40]. Therefore, the presence of two clearly differentiated kinetic phases suggests the existence of complexity (i.e., heterogeneity) in the conformational ensemble of NQO1_apo_, and plausibly, the significant population of at least two conformational substates with different HDX behavior, which may or may not significantly reequilibrate upon the intrinsic HDX step. Interestingly, although ligand binding affects these two kinetic phases (NQO1_holo_ and NQO1_dic_, see Figures S6 and S7 and Tables S1 and S2), both these phases still contribute to the HDX kinetics in these ligation states, suggesting that a certain degree of conformational heterogeneity remains upon ligand binding. Second, although in the EX2 scenario the overall kinetics depends on the intrinsic HDX rate constant, and therefore, on the individual backbone amides and their vicinity [40], some sort of correlated behavior at larger scales than small protein segments (e.g., secondary structure elements) is observed (Figure 4B and Figure S9). Consistent with the above-mentioned proposal of a stable core of NQO1_apo_ with highly dynamic FBS and DBS (simply made by analysis of %D after 3 h, Figure 3), these kinetic analyses suggest that secondary structure elements outside the stable core typically exchanged quite fast (i.e., with large burst phases and with k_slow_ often in the range of 10^−1^ to 10^−2^ s^−1^; Figure 4B and Figure S9).

### 3.3. FAD and Dicoumarol Binding Cause Large-Scale Changes in Protein Structural Dynamics 

FAD binding to NQO1_apo_ is known to cause significant overall changes in protein structure and dynamics: it increases the content in the ordered secondary structure, reduces the protein hydrodynamic volume, and substantially enhances protein stability and resistance towards proteolytic attack [9,14,22,34,35]. In addition, structural and biophysical analyses have shown that NQO1 must contain bound FAD in order to bind dicoumarol with high affinity [9,14]. We first compared the %D incorporated to NQO1_holo_ and NQO1_apo_ after 3 h of reaction (Figure 5A,B), observing some interesting changes upon FAD binding. Particularly large differences were observed in loop L1 (involved in the MMI and the FBS), loop L4 (involved in the MMI, the FBS and the DBS) and helix α5 (involved in the FBS). The stabilization observed for the MMI thus explains the increased thermostability of the NQO1 dimer upon FAD binding. The much lower structural dynamics of the FBS upon FAD binding is also consistent with an induced-fit mechanism, in which binding competent states (with high structural stability) are marginally populated in the absence of FAD, according to a recent proposal based on binding structure-thermodynamic relationships [34]. Interestingly, these results also imply that FAD is not only required for dicoumarol binding as a part of the DBS, but also that FAD binding modifies the dynamics of protein structural elements involved in the binding of the inhibitor (Figure 5C). It is worth noting that FAD binding also significantly slows down (3- to 5-fold) HDX of other regions such as sheet β6, helix α7 and loop L3 (Figure 6 and Figure S6, and Table S1), some of them not directly involved in the MMI, FBS or DBS.

Dicoumarol binding to NQO1_holo_ is also known to increase the protein ordered secondary structure, thermal stability and resistance of the N-terminal domain towards proteolysis [9,14]. According to this evidence, we observed that dicoumarol binding to NQO1_holo_ decreased the %D after 3 h to an even larger extent than FAD binding, and these effects seemed to propagate to more distant regions in the protein structure, extensively affecting regions involved in the MMI, the FBS and DBS (Figure 5). The regions affected by dicoumarol binding were actually the very same set of structural regions affected by FAD binding with the exception of helix α5 (Figure 5). In contrast to FAD, dicoumarol binding also caused a dramatic reduction in %D in sheet β4 and loop L5, which contain residues belonging to the MMI and the DBS (Figure 5). In addition, dicoumarol binding slowed down the HDX kinetics of helix α7 and loops L4 and L5 (Figure 6 and Figures S6 and S7, and Tables S1 and S2) by several orders of magnitude.

Clearly, the HDX kinetics of NQO1 in all three ligation states (NQO1_apo_, NQO1_holo_ and NQO1_dic_) were complex overall (Figure 6 and Figures S6 and S7). Therefore, it was not straightforward to provide a single metric from the global kinetic analysis reported so far in this work, even when a simple kinetic model was used. To quantify the effects of ligand binding (and potentially of mutations) on the structural dynamics of NQO1, we sought a single metric that would respond, at least semi-quantitatively, to the different types of change observed upon ligand binding (Figure 5 and Figure 6). We must note that these changes include a variety of effects on the amplitudes of the two kinetic phases (sometimes increasing, decreasing, not changing or even shifting between the amplitudes of the burst and the slow phases upon ligand binding) and also on the rate constant of the slow phase (in some cases, this phase was too fast or slow to be measured adequately) (see Tables S1 and S2). Notably, all these effects on the HDX kinetics can be simplified to a common effect: at least a few of the data in the time-dependent measurements differ from two samples when these samples are paired for a given time point (see Figure 6 for representative examples). Thus, we decided to simply calculate the difference between the two paired proteins species for a given time and protein segment, and for each segment, to average the three time points with a maximal difference in these time series. Indeed, this simple metric (called Δ%D_av_) was able to detect and rank the effects of ligand binding on NQO1 HDX due to changes in amplitudes and kinetic rate constants (Figure 6).

We compared again the behavior of NQO1_holo_ vs. NQO1_apo_ using Δ%D_av_ (Figure 7A and Figure S10A). The results showed that FAD binding substantially reduced the backbone dynamics of residues 11–20 (loop L1 and helix α1), 46–76 (loops L2 and L3, helix α7 and sheet β6), 103–113 (sheet β6 and loop L4), 149–165 (loop L6) and 191–211 (loop L7 and helix α5) (Figure 7A). These regions include most of the residues involved in the MMI and FBS, and also some belonging to the DBS (Figure 7A,B). When we evaluated the effect of dicoumarol binding, we observed further structural stabilization of all these regions (with the exception of residues 191–211) and a specific stabilization of two additional regions mostly involved in the MMI and DBS: a dramatic stabilization of the segment 120–141 (loop 5) and a moderate effect in the segment 223–240 (loops L8 and L9 and sheets β8 and β9).

### 3.4. Insights into Cooperative Effects upon FAD and Dicoumarol Binding from Analysis of Structural Dynamics

**Scheme 2:**
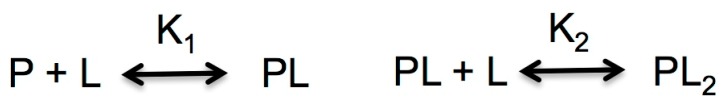
General formalism for a macromolecule P (NQO1 dimer) with two ligand (L) binding sites.

In addition to the overall conformational and functional consequences of FAD and dicoumarol binding to NQO1, some experimental techniques have revealed complexity in the functional chemistry of this enzyme [22,23,41]. This behavior is not unexpected since binding of FAD to NQO1_apo_ and dicoumarol to NQO1_holo_ is described by a general formalism for binding of a ligand (L) to a macromolecule (P, NQO1 dimer) with two binding sites (Scheme 2) [42]:

in which K_1_ and K_2_ describe the step-wise equilibrium constants as follows:

$$K_{1}=\frac{\left[PL\right]}{\left[P\right]\cdot \left[L\right]}$$

$$K_{2}=\frac{\left[PL_{2}\right]}{\left[PL\right]\cdot \left[L\right]}$$

For a P with two equivalent and non-interacting binding sites, these two step-wise equilibrium binding constants are related through a simple relationship: K_1_ = 4·K_2_. Significant deviations from this relationship imply the existence of non-equivalent or interacting sites: K_1_ > 4·K_2_ implies either negative cooperativity or non-equivalent binding sites, while K_1_ < 4·K_2_ unequivocally identifies positive cooperativity. It must be noted that, in this scenario, the largest differences between cooperative and non-cooperative binding are found for the dependence of the population of the half-ligated species [PL] (e.g., NQO1 dimer with one FAD bound) on [L] [22,23,42].

The NQO1 dimer contains two binding sites for either FAD or dicoumarol. Interestingly, calorimetric titrations of NQO1_apo_ with FAD and NQO1_holo_ with dicoumarol, as well as inhibition studies in the case of dicoumarol, have identified the existence of negative cooperativity in the binding of both ligands [22,23,41]. A detailed structural characterization of the communication between ligand binding sites underlying these cooperative effects is challenging for several reasons. First, in the ligand binding equilibrium, the unligated (P), half-ligated (PL) and fully-ligated (PL_2_) species contribute structurally and energetically to the observed cooperative binding. However, to date, no structural information on the P or PL states for FAD binding is available (i.e., no high-resolution structure of NQO1_apo_ or the intermediate species with a single FAD molecule bound per dimer), while for dicoumarol binding, no structural information for PL (NQO1_holo_ with a single dicoumarol molecule bound per dimer) is available. Although our current HDXMS study does not report on half-ligated species, we were able to identify a network of interacting residues in NQO1_apo_ that connect structurally and energetically the FBS and the DBS between monomers through the MMI interface. Therefore, our results suggest the existence of a highly dynamic structural network connecting these binding sites between the monomers in NQO1, and these results may also provide a blueprint for future experimental and computational mutagenesis studies aimed at perturbing and analyzing the role of this network in ligand binding energetics and cooperativity. In support of this hypothesis, recent work has shown that the mutation Gly151Ser (Gly150 according to the crystal structure) essentially prevents the communication of ligand binding effects between the two DBS across the NQO1 dimer [41]. Gly151 is located in the beginning of loop L6, and it undergoes a noticeable decrease in structural dynamics upon dicoumarol binding (Figure 7), which might constitute part of the allosteric signal generated by dicoumarol binding to one site (to form the half-ligated state PL), which communicates to the other subunit contributing to the negative cooperativity for dicoumarol binding. More globally, our analyses from HDXMS support the idea that dicoumarol binding triggers changes in NQO1 dynamics for certain (but not all) regions predicted by studies using a Gaussian network approach on structural models for the P and PL states [41]. In agreement with this recent study, we observed that dicoumarol binding affected the dynamics of loops L3 and L5, sheets β6 and β7, and helices α2, α5 and α7, which showed the largest changes in dynamics when the slowest frequency modes were analyzed upon formation of the PL state by a Gaussian network model (Figure 7 vs. [41]).

## 4. Conclusions

The multifunctional nature of NQO1 is likely controlled, to a large extent, by changes in protein structural dynamics triggered by the binding of small molecules (FAD, NAD(P)H, substrates, inhibitors) as well as by interaction with other biomacromolecules (proteins and nucleic acids). These structural and energetic aspects are critical to improve our understanding of these interactions under physiological and pathological conditions. In this work, we showed that the use of HDXMS can be instrumental to provide unprecedented detail of the effects of ligand binding on the functional chemistry of NQO1 linked to changes in protein structural dynamics. This approach may provide novel insights into the regulation of NQO1 activity and stability *in vivo*, as well as the mechanisms by which these properties are regulated and/or dysregulated by disease-associated single amino acid exchanges and post-translational modifications.

NQO1 is one of the human flavoproteins for which intracellular protein levels are more strongly coupled to the intracellular availability of the flavin cofactor [25]. Our current understanding of this phenomenon proposes that this sensitivity of protein stability is due to efficient recognition and degradation of human apo-flavoproteins by the ubiquitin-dependent proteasomal pathway [14,25,30]. The lack of high-resolution information on the structure and dynamics of human apo-flavoproteins, due to the instability of these apo-proteins, has prevented us from a deep understanding of these recognition mechanisms. The detailed analysis reported herein for NQO1_apo_ supports that HDXMS can also be used to improve our understanding of this phenomenon as well as the mechanisms by which disease-associated mutations and post-translational modifications may alter protein structural dynamics, leading to alternative recognition mechanisms by the proteasomal degradation pathway [14,30,34,36].

In this work, we identified a minimally stable core that allows NQO1_apo_ to exist as a dimer, although this dimer constitutes a highly dynamic conformational ensemble and with marginal conformational stability. This core may serve as a wiring network that allows the communication of ligand binding and mutational effects between domains and between subunits through this minimally stable MMI (see [9,22,23,32,33]), and cooperative effects upon FAD and dicoumarol binding [22,23,41].

Our study also allows us to discuss the deleterious effects of the common cancer-associated polymorphism Pro187Ser. Pro187 belongs to sheet β5, as a part of the stable core of the monomer in NQO1_apo_ state (Figure 3). Thus, the strong structural perturbation presumably caused by Pro187Ser substitution could readily cause long-range effects on the structural dynamics of NQO1_apo_ by disrupting this stable core, and these effects could propagate within the monomer and between subunits in the dimer. This interpretation agrees with previous experimental, computational and structural perturbation analyses carried out on the Pro187Ser variant [5,14,22,30,31,32,33,34,36]. Consequently, even in NQO1_apo_, the cancer-associated polymorphism Pro187Ser would destabilize the NQO1 monomer, and this effect could easily propagate to the MMI, the FBS and the DBS, thus contributing to explain the low conformational stability of P187S *in vitro*, its low affinity for FAD and dicoumarol, and its accelerated degradation by the proteasome [9,10,14,22,23,24,25,34].

Our analyses also support that HDXMS can provide unprecedented structural insight into the catalytic cycle of NQO1. First, our results indicate that FAD binding shifts the conformational ensemble of NQO1 towards more stable and competent states for either NAD(P)H or dicoumarol binding (i.e., at the DBS). This effect is further strengthened upon the binding of the inhibitor, suggesting that the structural dynamics of the DBS (and plausibly of the NAD(P)H binding site; [15]) acts by limiting the available ligand binding poses of NAD(P)H. This would optimize hydride transfer from the adenine dinucleotide coenzyme to the FAD, thus contributing to high rate constants experimentally measured for the reductive half-reaction catalyzed by NQO1 [10].

## Supplementary Materials

The following are available online at https://www.mdpi.com/2218-273X/9/11/728/s1. In the Supplementary information file, Figures S1–S11 and Tables S1 and S2 can be found.
