# Supplementary material for: A Dynamic Core in Human NQO1 Controls the Functional and Stability Effects of Ligand Binding and Their Communication across the Enzyme Dimer
Source: Biomolecules. 2019 Nov 12;9(11):728. doi: 10.3390/biom9110728 (PMC6921033; doi:10.3390/biom9110728)
Supplement: Supplementary file 1 [file biomolecules-09-00728-s001.pdf]

SUPPLEMENTARY INFORMATION

A dynamic core in human NQO1 controls the functional and stability effects of ligand binding and their communication across the enzyme dimer

Pavla Vankova, Eduardo Salido, David J. Timson, Petr Man and Angel L. Pey

Includes Figures S1-S11 and Tables S1-S2.

Figure S1. Purity and activity of the NQO1 protein. A) SDS-PAGE analysis (12 % acrylamide) of NQO1 protein purified upon expression in *E. coli*. Different gels show purified NQO1 protein (6-8 μg) as holo- and apo-proteins, respectively. Gels were stained with Coomassie® Brilliant blue R250 (Sigma-Aldrich). B) Enzyme kinetic analysis of holo-NQO1 in the presence of a fixed DCPIP concentration (20 μM) and variable concentrations of NADH. Prior to the assay, 1 nM holo-NQO1 was incubated in K-HEPES 50 mM pH 7.4 with NADH for 5 min at 25ºC and the reaction triggered by adding DCPIP. Blanks in the absence of enzyme were also measured and subtracted. The specific activity was determined spectrophotometrically essentially as described [1]. Each point represents the average of two replicates. Data were collected in three independent experimental series. The line is a fit to the Michaelis-Menten equation providing values of k_{cat} and $K_{M(NADH)}$ of 50±4 s^{-1} and 540±90μM, respectively.

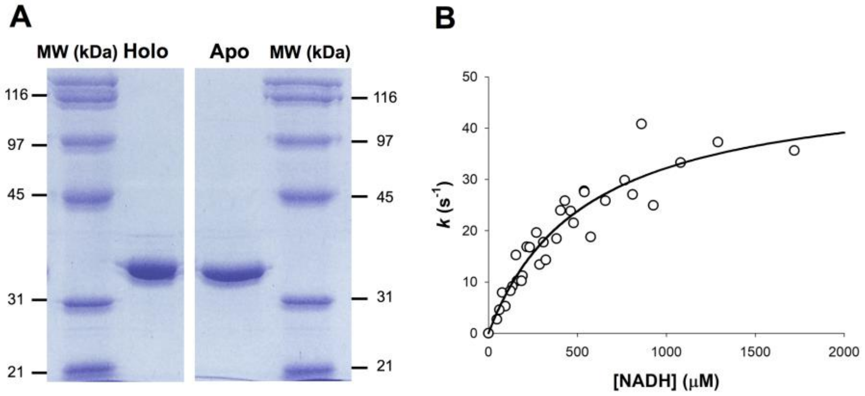

Figure S2. Verification of the NQO1 protein by high resolution mass spectrometry. NQO1 was offline desalted on a Protein OptiTrap (Optimize Technologies,). Then 5 μM sample was directly infused into an ESI source of 15T FT-ICR MS (solariX XR, Bruker Daltonics) and the spectra were recorded in a broad band mode with 2M data points. Spectrum shows the entire charge state envelope (detected charges 19+ to 48+) and inset demonstrates the high resolving power and isotopic pattern for charge state 32+. Spectrum was deconvoluted using the SNAP algorithm (DataAnalysis 5.0). Calculated monoisotopic mass and the experimental deconvoluted values are shown together with the error in ppm. The precise mass exactly fits to the sequence of the construct (shown below the spectrum) without the N-terminal methionine (shown in grey). Grey highlight shows the His-tag sequence. This is also indicated by the negative (non-native) numbering.

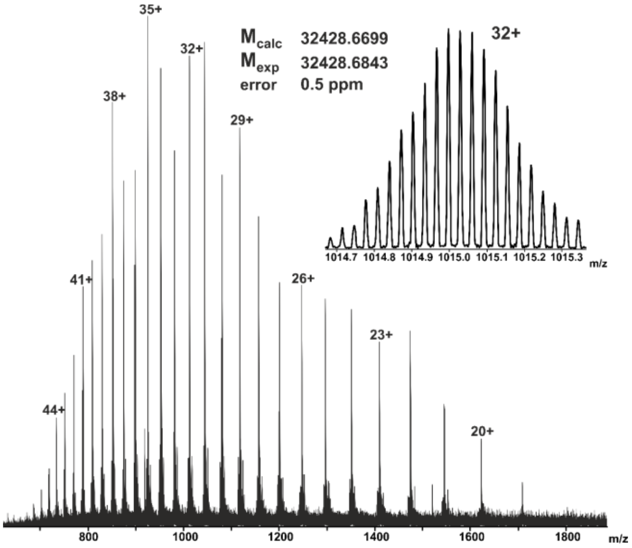


```
-14          -1     6         16         26         36
MAHHHHHHVD DDDKMVGRRA LIVLAHSERT SFNYAMKEAA AAALKKKGWE
        46         56         66         76         86
VVESDLYAMN FNPIISRKDI TGKLKDPANF QYPAESVLAY KEGHLSPDIV
        96        106        116        126        136
AEQKKLEAAD LVIFQFPLQW FGVPAILKGW FERVFIGEFA YTYAAMYDKG
       146        156        166        176        186
PFRSKKAVLS ITTGGSGSMY SLQGIHGDMN VILWPIQSGI LHFCGFQVLE
       196        206        216        226        236
PQLTYSIGHT PADARIQILE GWKKRLENIW DETPLYFAPS SLFDLNFQAG
       246        256        266      274
FLMKKEVQDE EKNKKFGLSV GHHLGKSIPT DNQIKARK
```

Figure S3. NQO1 dimeric state confirmed by mass spectrometry. $NQO1_{apo}$ (A) and $NQO1_{holo}$ (B) were transferred into 100 mM ammonium acetate pH 7.5 by Zeba spin (Thermo Fisher) desalting columns. Protein solution was transferred into a home-made quartz nESI emitter that was mounted to an nESI source of Synapt G2Si (Waters). Spray voltage was kept at 0.7 kV. Temperature was 20 °C, sampling cone and source offset were 50 V and 20 V, respectively. Trap collisional cooling (CE) of 80 V was required to achieve better S/N and resolution. The analyses proved that NQO1 forms stable dimer in both states. Charge states of individual peaks are shown. Dimeric protein state is indicated by two circles – open for $NQO1_{apo}$ and closed for $NQO1_{holo}$.

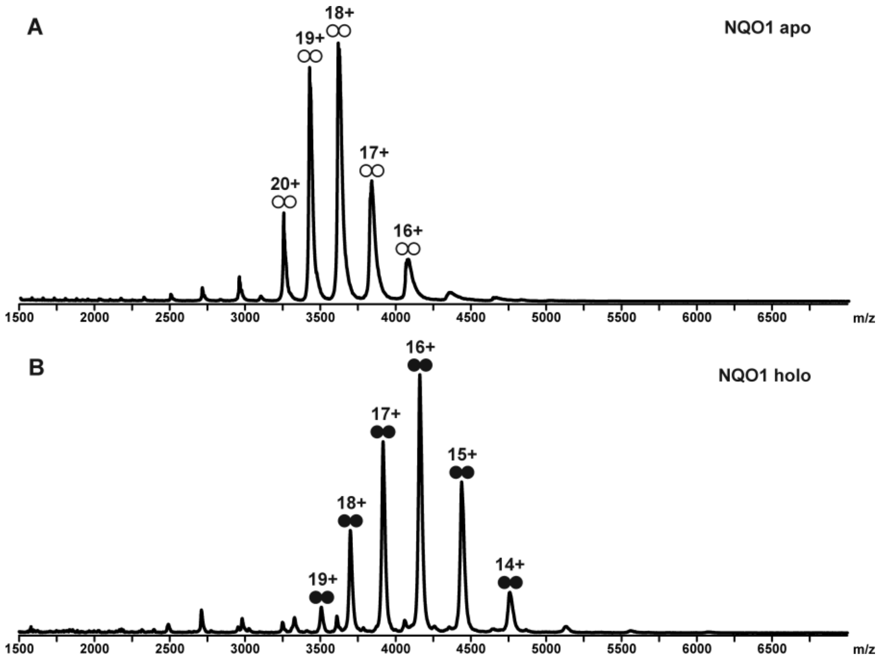

Figure S4. HDXMS mapping. NQO1 peptide map representing sequence coverage in the HDXMS study. Protein was digested online by a serial combination of Nepenthesin-2 and Pepsin. This yielded 140 peptides with the average peptide length 8.3 amino acids and redundancy score 4.05 covering nearly 99% of the sequence. Peptides were identified by LC-MS/MS analysis and MASCOT searching as described previously. Coverage is shown on the sequence of the construct with numbering reflecting native one. Secondary structure elements are shown as cylinders (alpha helices) and arrows (beta sheets). Numbering of loops is also indicated (L1-L10) above the sequence. The His-tag is highlighted as a grey box. Map was created using MSTools (http://peterslab.org/MSTools/DrawMap/DrawMap.php). [2].

Deleted: -

Deleted: -

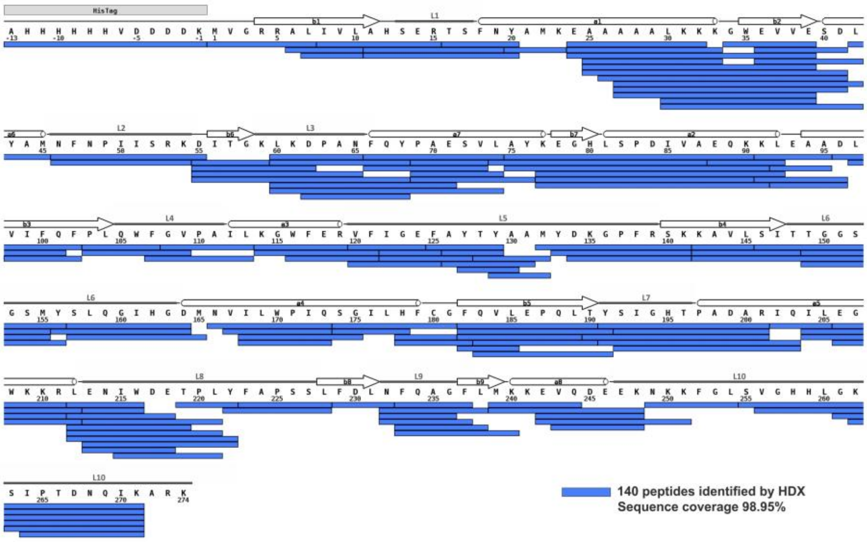

Figure S5. Representative examples of rare EX1/EX2 exchange profiles. The NQO1 protein showed predominantly EX2 exchange behavior in all ligation states as well as in the apo form. Exceptions were two regions covered by peptides 75-90, 77-90, 77-91, 77-92 and 249-254, 255-271, 256-271, 261-271, 262-271, 263-271, 264-271 where very small EX1 signatures were detected. Two representative examples are shown in this figure. A) Peptide 77-92 where top left graph shows peak width detected at 1% of the MS peak intensity as a function of time. Other three panels are showing isotopic envelopes of $NQO1_{apo}$, $NQO1_{holo}$ and $NQO1_{dic}$ at 300 s of exchange (position in the graph indicate this time point). B) peptide 255-271, peak width is again in the top left part of the panel and the other three panels show mass spectra of $NQO1_{apo}$, $NQO1_{holo}$ and $NQO1_{dic}$ at 10 s of exchange (position in the graph indicate this time point).

Deleted: for dicoumarol and wild-type states respectively.

Deleted: p

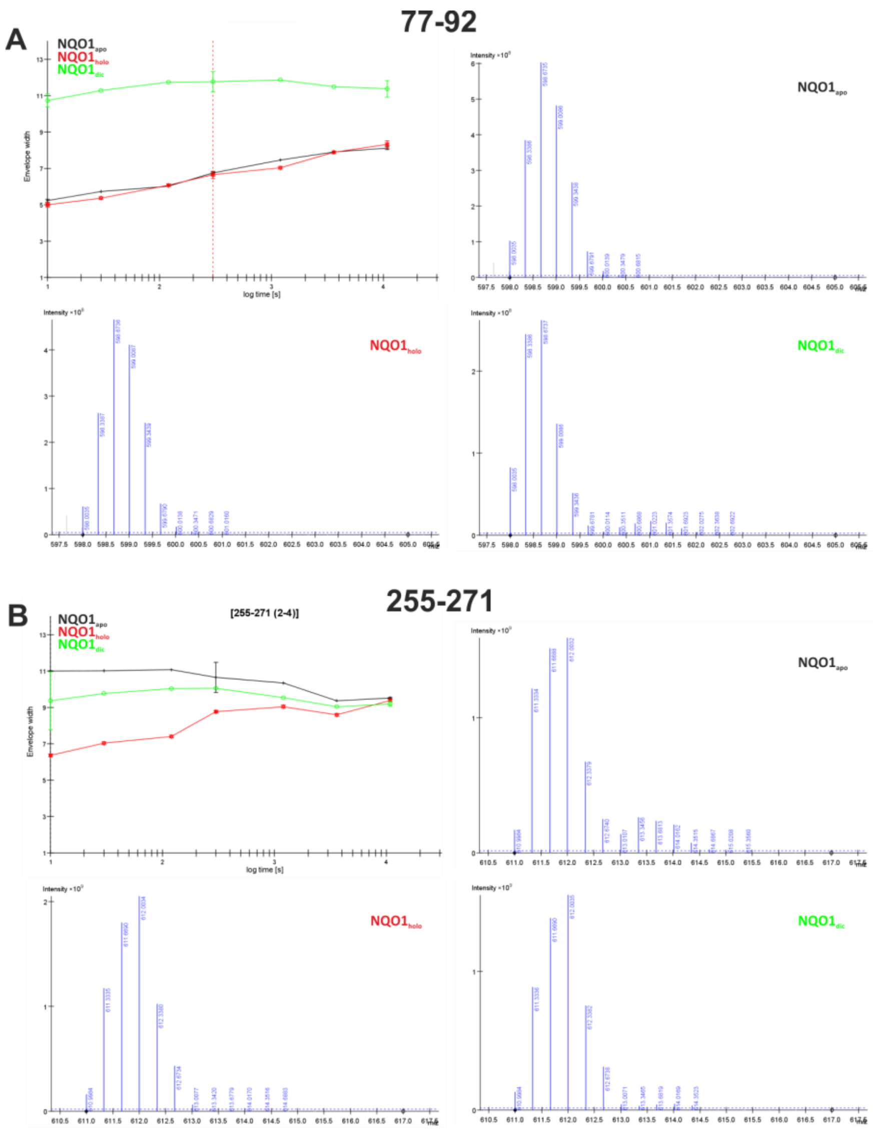
A
77-92
NQO1apo
NQO1holo
NQO1dic
Envelope width
log time [s]
B
255-271
[255-271 (2-4)]
NQO1apo
NQO1holo
NQO1dic
Envelope width
log time [s]

Figure S6 (1 of 3). HDX kinetics for NQO1 segments (spanning residues 1-95) of NQO1 upon FAD and dicoumarol binding. Plots show percentage of deuteration as a function of time. Segment limits are shown in each graph. Kinetic analysis can be found in Table S1.

Deleted: ing

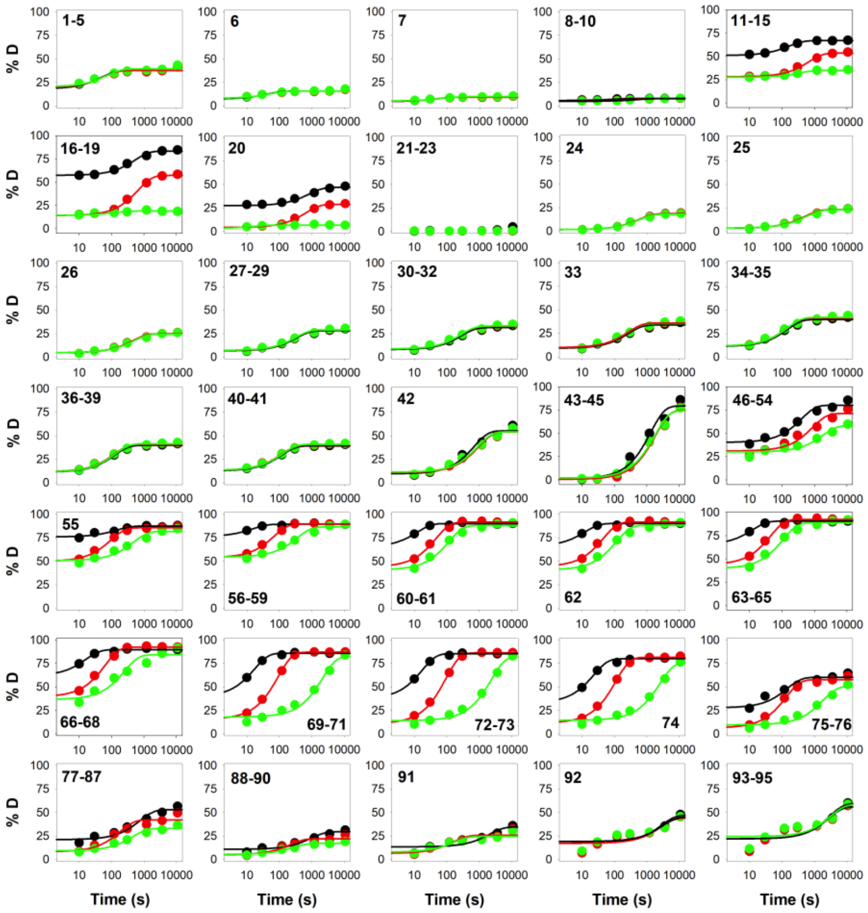

Figure S6 (2 of 3). HDX kinetics for NQO1 segments (spanning residues 96-181) of NQO1 upon FAD and dicoumarol binding. Plots show percentage of deuteration as a function of time. Segment limits are shown in each graph. Kinetic analysis can be found in Table S1.

Deleted: ing

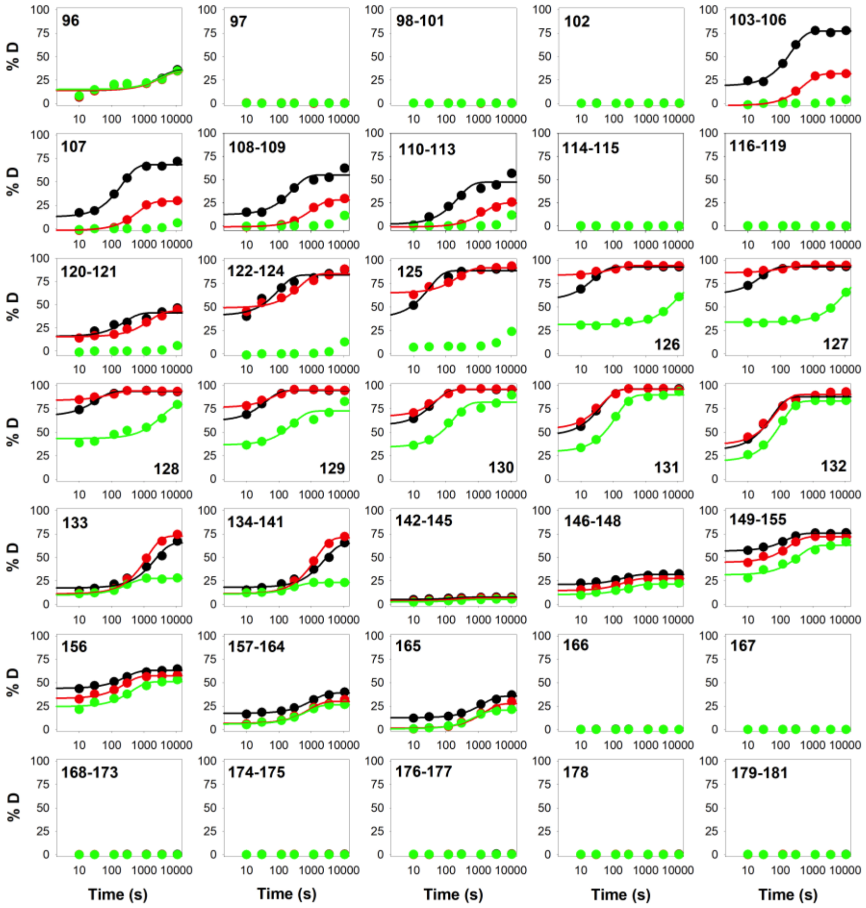

Figure S6 (3 of 3). HDX kinetics for NQO1 segments (spanning residues 182-271) of NQO1 upon FAD and dicoumarol binding. Plots show percentage of deuteration as a function of time. Segment limits are shown in each graph. Kinetic analysis can be found in Table S1.

Deleted: ing

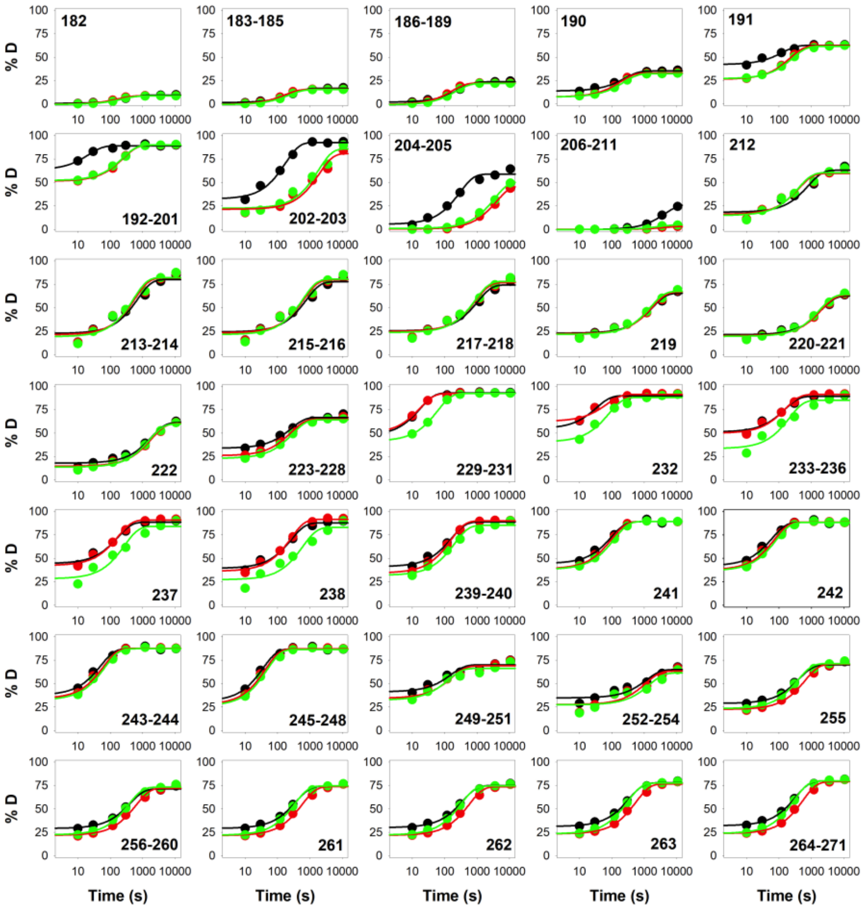

Figure S7. HDX kinetics for 39 non-overlapping and non-redundant peptides of NQO1 upon FAD and dicoumarol binding. Plots show percentage of deuteration as a function of time. Peptide limits are shown in each graph. These analyses are meant to be compared with those displayed in Figures S6. Kinetic analyses can be found in Table S2.

Deleted: ing

Deleted: 7-S9

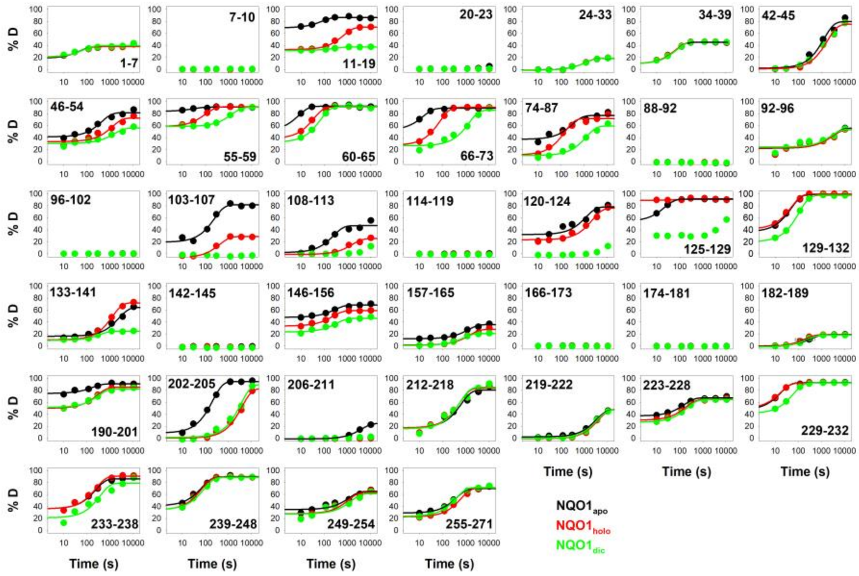

Figure S8. ***Non-exchanging*** **peptides define a minimal stable core in $NQO1_{apo}$.** A) Plot of the % SASA for individual residues (considering backbone and side-chain) calculated as indicated Figure 3. Secondary structure elements are depicted according to [3]; B) Structural representation of non-exchanging residues (using PDB 2F1O; [4]). The left panel shows a surface representation highlighting the burial of the minimal and stable core. The middle panel shows segments belonging to this core plotted onto secondary structures. The right panel shows that the core may contribute to the stable folding of the individual monomers as well as their assembly into the dimer, with only some stable contacts with the FAD (in orange ball representation) and the dicoumarol (in yellow ball representation). Note that this analysis for the 39 experimental peptides (Table S2) only shows small differences (a longer α1 helix and a *new* non-exchanging short segment in helix α2) *vs.* those carried out with NQO1 segments (Table S1 and Figure 3).

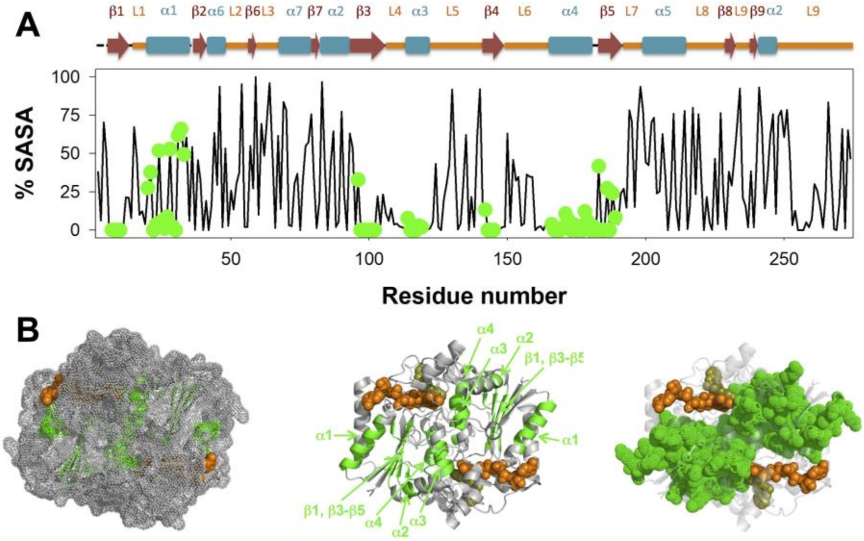

Figure S9. Specific HDX kinetics for 39 peptides of NQO1$_{apo}$. A) Plots of the amplitudes for the burst- and slow-phase in HDX for peptides (upper panel) and rate constant for the slow phase (lower panel) for peptides with at least 20% D after 3 h. The elements of secondary structure along the protein sequence are also indicated. Note that this analysis for the 39 experimental peptides (Table S2) show little differences when compared with those carried out with NQO1 segments (Table S1 and Figure 4).

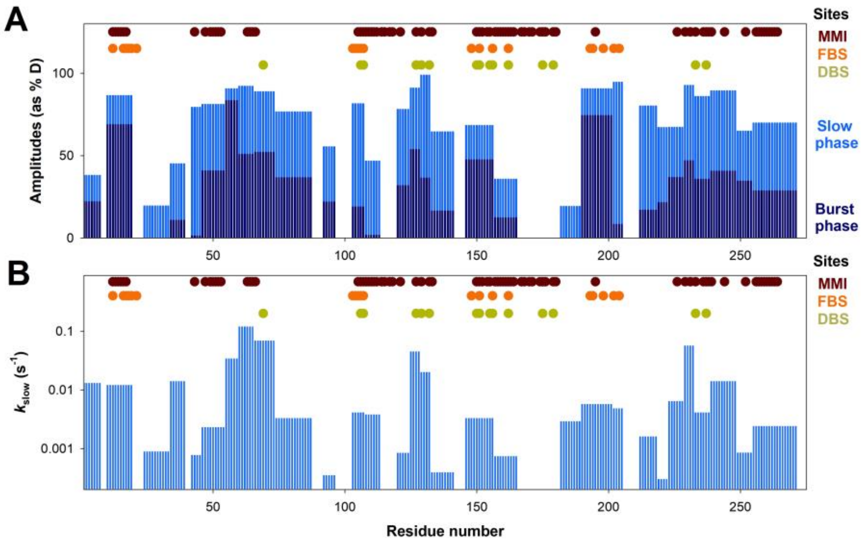

Figure S10. Changes in HDX kinetics for 39 peptides of NQO1 upon binding FAD and dicoumarol as changes in %D_{av} (Δ%D_{av}). A) Δ%D_{av} for peptides upon binding FAD ($NQO1_{holo}$) and dicoumarol ($NQO1_{dic}$) using of $NQO1_{apo}$ as a reference (see Figure 7 for details). B) Representation of (Δ%D_{av}) onto the structure of NQO1 (using PDB code 2F1O). The upper row shows the results for Δ%D_{av} for $NQO1_{holo}$ and lower row represents $NQO1_{dic}$. Different panels in each row show results for residues involved in the FBS, DBS or MMI. Note that this analysis for 39 experimental peptides (Table S2) shows little differences with those data carried out with NQO1 segments (Table S1 and Figure 7).

Deleted: the 105 segments

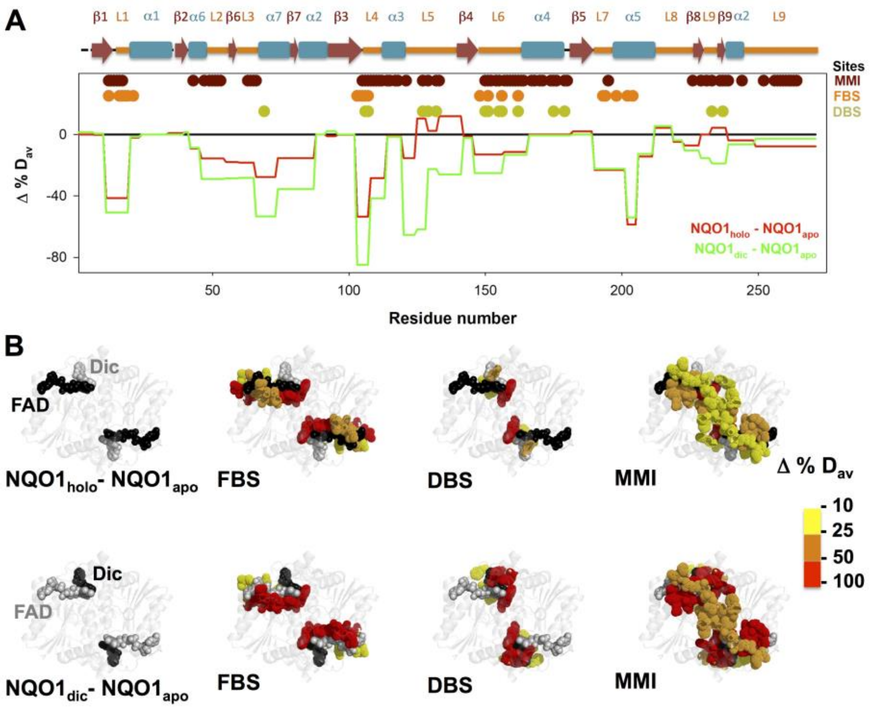

Figure S11 (three pages). HDX kinetics for all peptides detected in HDXMS experiments. Each peptide is represented by one deuterium uptake plot where number of uptaken deuterons is plotted as a function of time. Black stands for $NQO1_{apo}$, red for $NQO1_{holo}$ and green for $NQO1_{dic}$. Times points followed during experiment were 10 s, 30 s, 2 min, 5 min, 20 min, 1 h and 3 h. Labeling and analysis at 10 s, 5 min and 3 h was replicated and the data are shown as average values with standard deviation (error bars at these points). Peptide limits are shown at the top of each graph together with the range or charge states that were detected for the peptide.

Deleted: -

Deleted: retained

Deleted: s

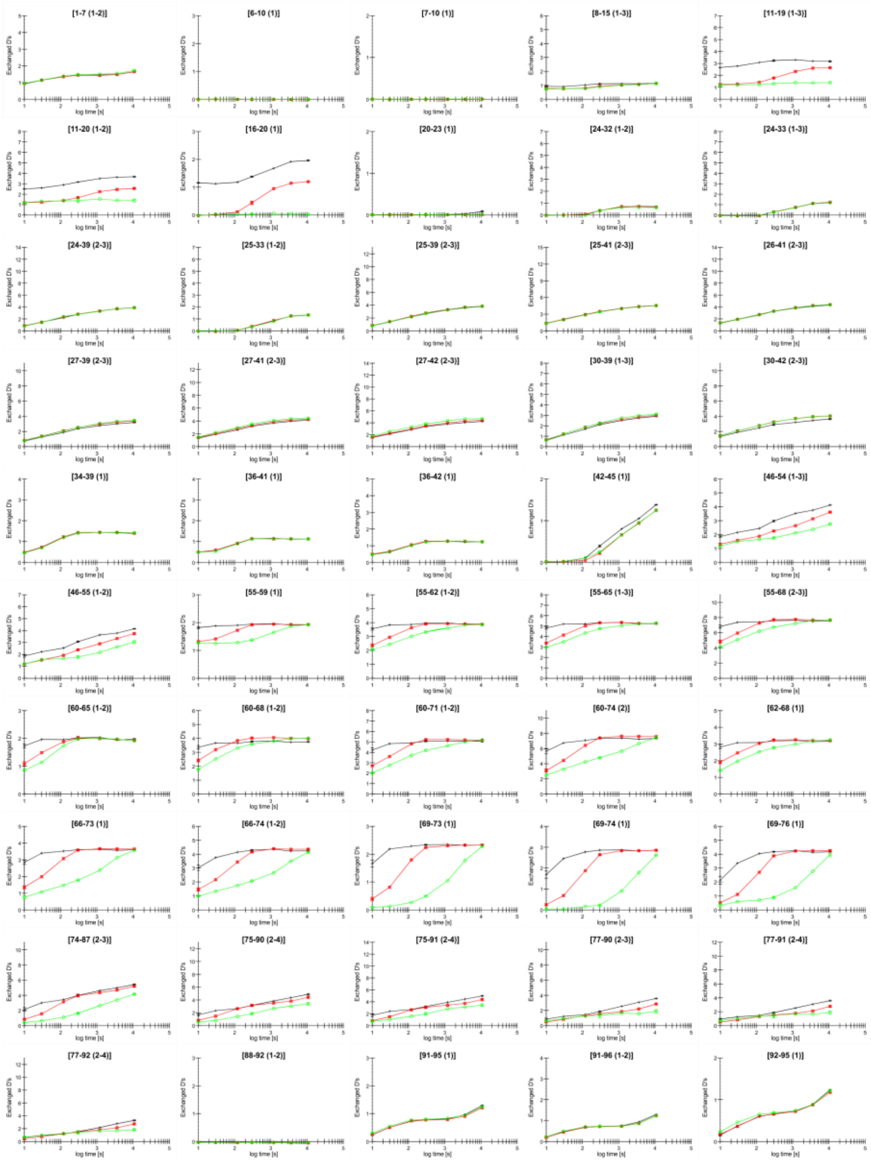
[1-7 (1-2)]
[6-10 (1)]
[7-10 (1)]
[8-15 (1-3)]
[11-19 (1-3)]
[11-20 (1-2)]
[16-20 (1)]
[20-23 (1)]
[24-32 (1-2)]
[24-33 (1-3)]
[24-39 (2-3)]
[25-33 (1-2)]
[25-39 (2-3)]
[25-41 (2-3)]
[26-41 (2-3)]
[27-39 (2-3)]
[27-41 (2-3)]
[27-42 (2-3)]
[30-39 (1-3)]
[30-42 (2-3)]
[34-39 (1)]
[36-41 (1)]
[36-42 (1)]
[42-45 (1)]
[46-54 (1-3)]
[46-55 (1-2)]
[55-59 (1)]
[55-62 (1-2)]
[55-65 (1-3)]
[55-68 (2-3)]
[60-65 (1-2)]
[60-68 (1-2)]
[60-71 (1-2)]
[60-74 (2)]
[62-68 (1)]
[66-73 (1)]
[66-74 (1-2)]
[69-73 (1)]
[69-74 (1)]
[69-76 (1)]
[74-87 (2-3)]
[75-90 (2-4)]
[75-91 (2-4)]
[77-90 (2-3)]
[77-91 (2-4)]
[77-92 (2-4)]
[88-92 (1-2)]
[91-95 (1)]
[91-96 (1-2)]
[92-95 (1)]
Exchanged D's
log time [s]

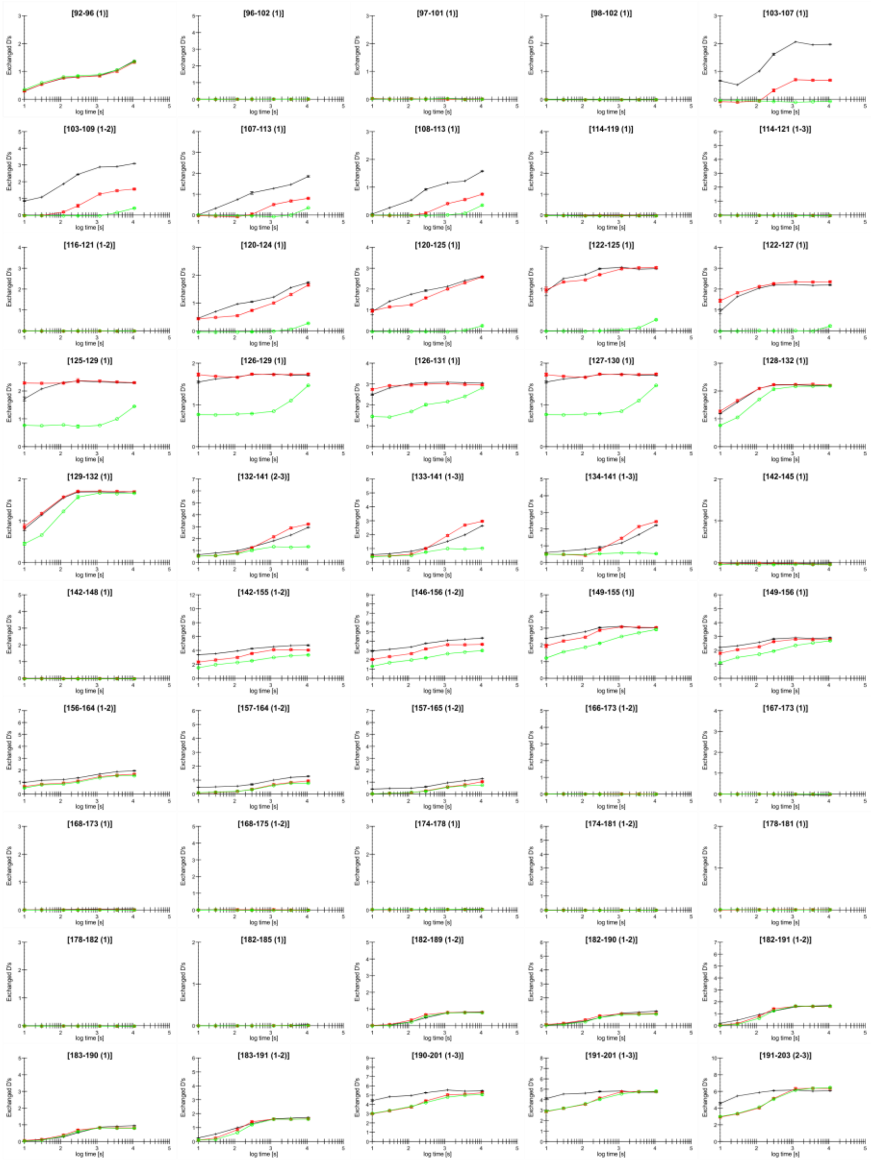
[92-96 (1)]
[96-102 (1)]
[97-101 (1)]
[98-102 (1)]
[103-107 (1)]
[103-109 (1-2)]
[107-113 (1)]
[108-113 (1)]
[114-119 (1)]
[114-121 (1-3)]
[116-121 (1-2)]
[120-124 (1)]
[120-125 (1)]
[122-125 (1)]
[122-127 (1)]
[125-129 (1)]
[126-129 (1)]
[126-131 (1)]
[127-130 (1)]
[128-132 (1)]
[129-132 (1)]
[132-141 (2-3)]
[133-141 (1-3)]
[134-141 (1-3)]
[142-145 (1)]
[142-148 (1)]
[142-155 (1-2)]
[146-156 (1-2)]
[149-155 (1)]
[149-156 (1)]
[156-164 (1-2)]
[157-164 (1-2)]
[157-165 (1-2)]
[166-173 (1-2)]
[167-173 (1)]
[168-173 (1)]
[168-175 (1-2)]
[174-178 (1)]
[174-181 (1-2)]
[178-181 (1)]
[178-182 (1)]
[182-185 (1)]
[182-189 (1-2)]
[182-190 (1-2)]
[182-191 (1-2)]
[183-190 (1)]
[183-191 (1-2)]
[190-201 (1-3)]
[191-201 (1-3)]
[191-203 (2-3)]
Exchanged D's
log time [s]

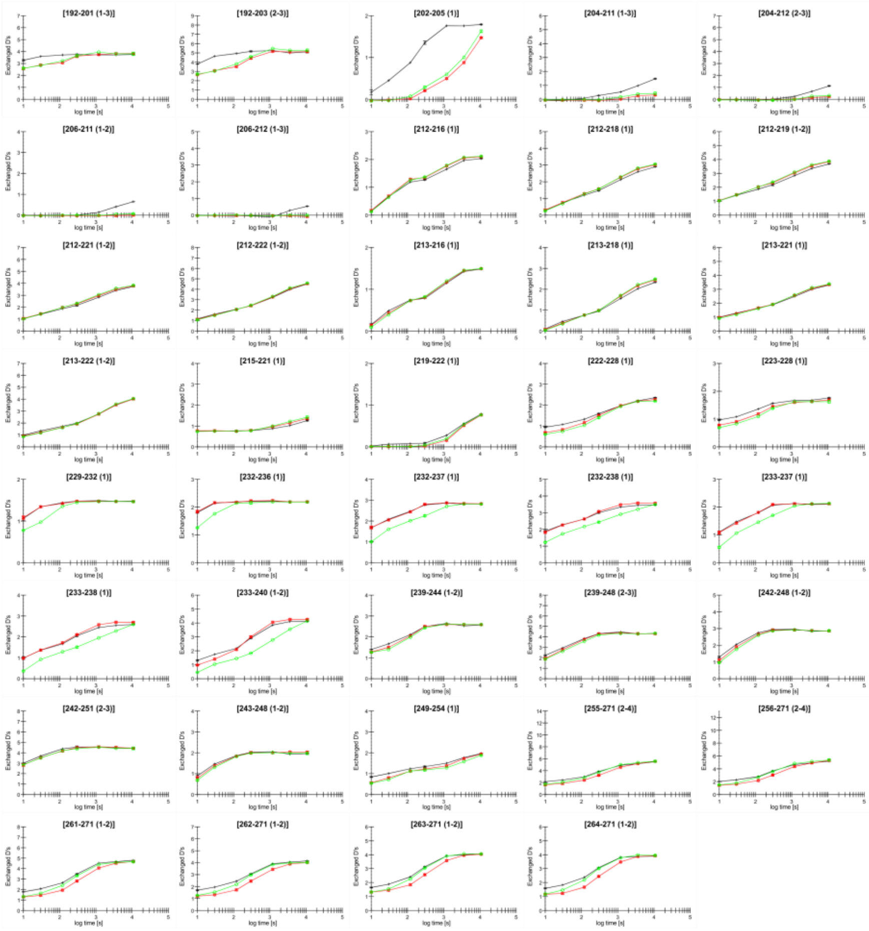
[192-201 (1-3)]
[192-203 (2-3)]
[202-205 (1)]
[204-211 (1-3)]
[204-212 (2-3)]
[206-211 (1-2)]
[206-212 (1-3)]
[212-216 (1)]
[212-218 (1)]
[212-219 (1-2)]
[212-221 (1-2)]
[212-222 (1-2)]
[213-216 (1)]
[213-218 (1)]
[213-221 (1)]
[213-222 (1-2)]
[215-221 (1)]
[219-222 (1)]
[222-228 (1)]
[223-228 (1)]
[229-232 (1)]
[232-236 (1)]
[232-237 (1)]
[232-238 (1)]
[233-237 (1)]
[233-238 (1)]
[233-240 (1-2)]
[239-244 (1-2)]
[239-248 (2-3)]
[242-248 (1-2)]
[242-251 (2-3)]
[243-248 (1-2)]
[249-254 (1)]
[255-271 (2-4)]
[256-271 (2-4)]
[261-271 (1-2)]
[262-271 (1-2)]
[263-271 (1-2)]
[264-271 (1-2)]
Exchanged D's
log time [s]

Table S1. Kinetic parameters for fitting of HDX kinetics to a three-parameter exponential function for NQO1 segments. *SLNE* indicates *slow, little or no exchange*.

Segment	NQO1 protein	A_{burst} (%D)	A_{slow} (%D)	k_{slow} (s^{-1})	R^2
1-5	$NQO1_{apo}$	22.3±2.7	15.8±2.8	$1.2±0.6 \cdot 10^{-2}$	0.914
	$NQO1_{holo}$	23.0±2.8	15.0±2.8	$1.2±0.6 \cdot 10^{-2}$	0.898
	$NQO1_{dic}$	23.5±2.6	16.2±2.7	$1.0±0.5 \cdot 10^{-2}$	0.912
6	$NQO1_{apo}$	8.8±1.3	7.0±1.3	$1.5±0.8 \cdot 10^{-2}$	0.916
	$NQO1_{holo}$	9.6±1.2	6.3±1.2	$1.2±0.7 \cdot 10^{-2}$	0.893
	$NQO1_{dic}$	9.5±1.2	7.0±1.2	$1.1±0.5 \cdot 10^{-2}$	0.912
7	$NQO1_{apo}$	5.2±0.7	4.0±0.7	$1.4±0.7 \cdot 10^{-2}$	0.915
	$NQO1_{holo}$	5.5±0.7	3.7±0.7	$1.2±0.7 \cdot 10^{-2}$	0.893
	$NQO1_{dic}$	5.6±0.6	4.1±0.6	$1.0±0.4 \cdot 10^{-2}$	0.921
8-10	$NQO1_{apo}$	6.1±0.1	1.7±0.1	$7.7±1.9 \cdot 10^{-3}$	0.973
	$NQO1_{holo}$	5.3±0.2	2.3±0.2	$1.9±0.7 \cdot 10^{-3}$	0.957
	$NQO1_{dic}$	5.0±0.2	2.7±0.3	$1.6±0.5 \cdot 10^{-3}$	0.966
11-15	$NQO1_{apo}$	51.0±0.3	15.9±0.4	$6.0±0.4 \cdot 10^{-3}$	0.998
	$NQO1_{holo}$	28.1±0.7	25.5±1.0	$1.5±0.2 \cdot 10^{-3}$	0.994
	$NQO1_{dic}$	27.5±0.4	7.6±0.5	$2.7±0.6 \cdot 10^{-3}$	0.983
16-19	$NQO1_{apo}$	57.4±1.0	26.0±1.3	$1.9±0.3 \cdot 10^{-3}$	0.990
	$NQO1_{holo}$	14.6±0.8	42.8±1.1	$1.5±0.1 \cdot 10^{-3}$	0.997
	$NQO1_{dic}$	15.4±0.7	3.6±0.8	$5.2±3.2 \cdot 10^{-3}$	0.845
20	$NQO1_{apo}$	28.7±0.6	18.5±0.9	$1.0±0.2 \cdot 10^{-3}$	0.990
	$NQO1_{holo}$	4.5±0.5	24.5±0.6	$1.5±0.1 \cdot 10^{-3}$	0.997
	$NQO1_{dic}$	5.2±0.6	1.7±0.6	$1.0±1.1 \cdot 10^{-2}$	0.695
21-23	$NQO1_{apo}$	SLNE			
	$NQO1_{holo}$	SNLE			
	$NQO1_{dic}$	SNLE			
24	$NQO1_{apo}$	1.7±0.7	17.0±0.8	$1.7±0.3 \cdot 10^{-3}$	0.990

	$NQO1_{holo}$	1.8±0.6	16.9±0.8	$1.8±0.3 \cdot 10^{-3}$	0.992
	$NQO1_{dic}$	1.7±0.7	16.4±0.8	$1.8±0.3 \cdot 10^{-3}$	0.989
25	$NQO1_{apo}$	3.7±1.0	19.5±1.3	$1.9±0.4 \cdot 10^{-3}$	0.983
	$NQO1_{holo}$	3.7±1.0	19.5±1.3	$1.9±0.4 \cdot 10^{-3}$	0.984
	$NQO1_{dic}$	3.8±1.0	19.2±1.3	$1.8±0.4 \cdot 10^{-3}$	0.982
26	$NQO1_{apo}$	4.6±1.2	20.0±1.5	$2.2±0.5 \cdot 10^{-3}$	0.979
	$NQO1_{holo}$	4.7±1.2	20.1±1.4	$2.2±0.5 \cdot 10^{-3}$	0.980
	$NQO1_{dic}$	4.7±1.2	19.8±1.5	$2.1±0.5 \cdot 10^{-3}$	0.977
27-29	$NQO1_{apo}$	6.9±1.5	21.2±1.8	$2.9±0.5 \cdot 10^{-3}$	0.974
	$NQO1_{holo}$	7.3±1.5	21.5±1.8	$3.0±0.7 \cdot 10^{-3}$	0.975
	$NQO1_{dic}$	7.4±1.5	21.6±1.9	$2.9±0.7 \cdot 10^{-3}$	0.973
30-32	$NQO1_{apo}$	8.3±1.7	23.1±2.1	$3.4±0.9 \cdot 10^{-3}$	0.970
	$NQO1_{holo}$	9.0±1.8	23.7±2.1	$3.5±0.9 \cdot 10^{-3}$	0.972
	$NQO1_{dic}$	9.1±1.8	24.1±2.2	$3.5±0.9 \cdot 10^{-3}$	0.971
33	$NQO1_{apo}$	9.5±2.0	24.4±2.4	$3.5±1.0 \cdot 10^{-3}$	0.965
	$NQO1_{holo}$	10.3±2.1	25.7±2.5	$3.5±1.0 \cdot 10^{-3}$	0.964
	$NQO1_{dic}$	10.4±2.1	25.7±2.3	$3.5±1.0 \cdot 10^{-3}$	0.963
34-35	$NQO1_{apo}$	12.3±2.1	27.8±2.3	$6.4±1.5 \cdot 10^{-3}$	0.975
	$NQO1_{holo}$	13.0±2.1	28.6±2.4	$6.7±1.5 \cdot 10^{-3}$	0.976
	$NQO1_{dic}$	12.9±2.2	29.4±2.5	$6.4±1.5 \cdot 10^{-3}$	0.974
36-39	$NQO1_{apo}$	12.5±1.5	27.4±1.6	$7.5±1.2 \cdot 10^{-3}$	0.988
	$NQO1_{holo}$	13.1±1.5	27.9±1.7	$7.9±1.3 \cdot 10^{-3}$	0.987
	$NQO1_{dic}$	12.9±1.6	28.6±1.7	$7.3±1.2 \cdot 10^{-3}$	0.988
40-41	$NQO1_{apo}$	12.6 ±1.4	26.7±1.5	$8.3±1.3 \cdot 10^{-3}$	0.988
	$NQO1_{holo}$	13.3±1.5	27.1±1.6	$8.4±1.4 \cdot 10^{-3}$	0.988
	$NQO1_{dic}$	13.3±1.4	27.7±1.5	$7.9±1.2 \cdot 10^{-3}$	0.989
42	$NQO1_{apo}$	9.5±3.4	45.7±4.6	$1.4±0.5 \cdot 10^{-3}$	0.961
	$NQO1_{holo}$	10.8±2.6	43.3±3.8	$1.0±0.3 \cdot 10^{-3}$	0.971
	$NQO1_{dic}$	11.3±2.7	42.9±3.9	$1.1±0.3 \cdot 10^{-3}$	0.969

43-45	$NQO1_{apo}$	1.6±3.6	77.8±6.7	$7.7±1.8·10^{-4}$	0.987
	$NQO1_{holo}$	0.6±2.7	74.4±4.7	$5.4±1.1·10^{-4}$	0.987
	$NQO1_{dic}$	2.0±2.3	72.2±4.2	$5.7±1.0·10^{-4}$	0.988
46-54	$NQO1_{apo}$	40.4±3.1	39.9±3.8	$2.5±0.8·10^{-3}$	0.966
	$NQO1_{holo}$	31.1±3.6	40.2±5.1	$1.1±0.5·10^{-3}$	0.940
	$NQO1_{dic}$	30.0±2.1	28.4±3.9	$5.4±2.3·10^{-4}$	0.939
55	$NQO1_{apo}$	75.9±1.5	11.6±1.7	$6.7±2.7·10^{-3}$	0.930
	$NQO1_{holo}$	50.0±1.7	35.7±1.8	$1.0±0.1·10^{-2}$	0.992
	$NQO1_{dic}$	50.8±2.7	31.0±3.4	$2.1±0.8·10^{-3}$	0.954
56-59	$NQO1_{apo}$	76.3±4.9	13.1±4.8	$6.5±3.1·10^{-2}$	0.898
	$NQO1_{holo}$	53.8±1.2	36.1±1.2	$1.4±0.1·10^{-2}$	0.997
	$NQO1_{dic}$	54.5±2.7	32.1±3.2	$2.8±0.9·10^{-3}$	0.963
60-61	$NQO1_{apo}$	65.2±8.8	24.9±8.7	$8.1±3.2·10^{-2}$	0.938
	$NQO1_{holo}$	44.0±2.8	47.7±2.7	$2.2±0.3·10^{-2}$	0.993
	$NQO1_{dic}$	40.9±3.6	47.1±3.8	$8.7±2.0·10^{-3}$	0.978
62	$NQO1_{apo}$	66.0±8.6	24.2±8.5	$8.0±3.2·10^{-2}$	0.938
	$NQO1_{holo}$	44.4±2.8	47.5±2.8	$2.2±0.3·10^{-2}$	0.993
	$NQO1_{dic}$	40.9±3.9	47.3±4.1	$8.7±2.1·10^{-3}$	0.974
63-65	$NQO1_{apo}$	65.2±8.9	25.4±8.8	$8.0±3.2·10^{-2}$	0.938
	$NQO1_{holo}$	43.9±2.9	48.8±2.9	$2.2±0.3·10^{-2}$	0.993
	$NQO1_{dic}$	39.8±4.0	49.1±4.2	$9.2±2.2·10^{-3}$	0.975
66-68	$NQO1_{apo}$	61.8±5.5	27.9±5.5	$6.2±1.6·10^{-2}$	0.966
	$NQO1_{holo}$	39.5±2.4	53.0±2.4	$1.6±0.2·10^{-2}$	0.995
	$NQO1_{dic}$	36.6±5.9	47.6±6.9	$4.0±1.7·10^{-3}$	0.926
69-71	$NQO1_{apo}$	39.5±4.6	46.4±4.6	$5.8±0.8·10^{-2}$	0.991
	$NQO1_{holo}$	15.7±1.0	71.3±1.0	$1.1±0.1·10^{-2}$	0.999
	$NQO1_{dic}$	18.0±2.5	64.7±4.9	$4.7±1.0·10^{-4}$	0.982
72-73	$NQO1_{apo}$	37.2±4.6	48.2±4.5	$5.8±0.7·10^{-2}$	0.991
	$NQO1_{holo}$	11.0±1.5	75.1±1.5	$1.1±0.1·10^{-2}$	0.998

	NQO1$_{dic}$	14.2±2.2	68.1±4.5	4.3±0.8·10^{-4}	0.968
74	NQO1$_{apo}$	33.1±6.7	46.9±6.7	4.3±1.0·10^{-2}	0.975
	NQO1$_{holo}$	11.5±1.2	69.9±1.3	9.1±0.5·10^{-3}	0.999
	NQO1$_{dic}$	14.5±2.0	61.5±4.1	4.0±0.8·10^{-4}	0.986
75-76	NQO1$_{apo}$	30.2±4.3	30.3±4.8	5.9±2.6·10^{-3}	0.914
	NQO1$_{holo}$	7.2±3.0	50.4±3.2	7.4±1.3·10^{-3}	0.985
	NQO1$_{dic}$	9.9±2.0	40.7±3.7	5.4±1.5·10^{-4}	0.975
77-87	NQO1$_{apo}$	22.2±2.6	31.1±3.7	1.2±0.5·10^{-3}	0.946
	NQO1$_{holo}$	9.0±4.4	33.4±5.0	5.2±2.2·10^{-3}	0.922
	NQO1$_{dic}$	9.8±2.1	23.5±2.7	2.0±0.8·10^{-3}	0.951
88-90	NQO1$_{apo}$	10.9±1.5	18.7±2.3	1.0±0.4 ·10^{-3}	0.946
	NQO1$_{holo}$	5.0±2.6	17.2±3.0	4.6±2.3 ·10^{-3}	0.894
	NQO1$_{dic}$	5.4±1.2	11.9±1.4	3.5±1.3·10^{-3}	0.948
91	NQO1$_{apo}$	13.5±2.7	21.2±5.3	4.9±3.6 ·10^{-4}	0.831
	NQO1$_{holo}$	6.6±4.5	19.2±5.0	6.3±4.5 ·10^{-3}	0.803
	NQO1$_{dic}$	7.9±3.8	16.6±4.3	6.0±4.3·10^{-3}	0.805
92	NQO1$_{apo}$	17.3±3.9	30.0±8.8	3.6±3.0 ·10^{-4}	0.803
	NQO1$_{holo}$	17.4±4.0	27.0±8.6	3.8±3.5 ·10^{-4}	0.769
	NQO1$_{dic}$	19.2±3.8	26.1±8.3	3.3±3.1·10^{-4}	0.763
93-95	NQO1$_{apo}$	22.0±5.0	37.8±11.7	3.4±2.9 ·10^{-4}	0.791
	NQO1$_{holo}$	22.2±5.0	34.2±11.1	3.6±3.4 ·10^{-4}	0.764
	NQO1$_{dic}$	24.5±4.8	34.0±11.7	3.2±3.0·10^{-4}	0.764
96	NQO1$_{apo}$	14.0±2.8	22.0±6.6	3.2±2.7·10^{-4}	0.803
	NQO1$_{holo}$	14.2±2.8	20.4±7.1	3.0±2.8·10^{-4}	0.772
	NQO1$_{dic}$	15.3±2.7	19.9±7.1	2.9±2.8·10^{-4}	0.770
97	NQO1$_{apo}$	SLNE			
	NQO1$_{holo}$	SLNE			
	NQO1$_{dic}$	SLNE			
98-101	NQO1$_{apo}$	SLNE			

	$NQO1_{holo}$	SLNE			
	$NQO1_{dic}$	SLNE			
102	$NQO1_{apo}$	SLNE			
	$NQO1_{holo}$	SLNE			
	$NQO1_{dic}$	SLNE			
103-106	$NQO1_{apo}$	18.9±2.0	58.3±2.3	$4.5\pm0.5\cdot10^{-3}$	0.994
	$NQO1_{holo}$	-3.3±1.1	35.0±1.4	$2.0\pm0.3\cdot10^{-3}$	0.994
	$NQO1_{dic}$	SLNE			
107	$NQO1_{apo}$	13.8±2.0	54.7±2.3	$4.4\pm0.5\cdot10^{-3}$	0.994
	$NQO1_{holo}$	-2.6±0.9	31.8±1.2	$1.6\pm0.2\cdot10^{-3}$	0.995
	$NQO1_{dic}$	SLNE			
108-109	$NQO1_{apo}$	12.2±3.9	42.8±4.6	$3.6\pm1.2\cdot10^{-3}$	0.958
	$NQO1_{holo}$	-1.4±0.9	29.3±1.4	$9.7\pm1.5\cdot10^{-4}$	0.991
	$NQO1_{dic}$	SLNE			
110-113	$NQO1_{apo}$	1.8±5.3	45.4±6.2	$4.1\pm1.6\cdot10^{-3}$	0.934
	$NQO1_{holo}$	-1.1±1.1	25.9±1.9	$6.4\pm1.5\cdot10^{-3}$	0.980
	$NQO1_{dic}$	SLNE			
114-115	$NQO1_{apo}$	SLNE			
	$NQO1_{holo}$	SLNE			
	$NQO1_{dic}$	SLNE			
116-119	$NQO1_{apo}$	SLNE			
	$NQO1_{holo}$	SLNE			
	$NQO1_{dic}$	SLNE			
120-121	$NQO1_{apo}$	17.2±3.9	24.4±4.7	$3.2\pm1.9\cdot10^{-3}$	0.876
	$NQO1_{holo}$	16.1±1.4	27.2±2.5	$6.3\pm1.8\cdot10^{-4}$	0.972
	$NQO1_{dic}$	SLNE			
122-124	$NQO1_{apo}$	41.2±6.6	43.1±7.0	$9.8\pm4.5\cdot10^{-3}$	0.919
	$NQO1_{holo}$	49.3±3.5	36.4±4.4	$2.2\pm0.9\cdot10^{-3}$	0.946
	$NQO1_{dic}$	SLNE			

125	$NQO1_{apo}$	38.7±8.9	50.2±8.8	$3.2±1.1·10^{-2}$	0.958
	$NQO1_{holo}$	65.2±2.4	26.7±2.8	$4.1±1.2·10^{-3}$	0.961
	$NQO1_{dic}$	SLNE			
126	$NQO1_{apo}$	57.6±3.4	35.3±3.4	$4.0±0.7·10^{-2}$	0.988
	$NQO1_{holo}$	84.1±1.0	10.3±1.1	$9.6±2.8·10^{-3}$	0.965
	$NQO1_{dic}$	31.4±0.7	41.3±7.3	$1.6±0.4·10^{-4}$	0.991
127	$NQO1_{apo}$	63.8±3.3	29.4±3.3	$3.8±0.7·10^{-2}$	0.984
	$NQO1_{holo}$	86.7±0.7	8.0±0.8	$7.4±2.0·10^{-2}$	0.967
	$NQO1_{dic}$	33.9±0.5	45.3±6.0	$1.1±03 ·10^{-4}$	0.995
128	$NQO1_{apo}$	67.7±2.3	26.0±2.3	$3.0±0.5·10^{-2}$	0.988
	$NQO1_{holo}$	84.3±0.8	10.0±0.8	$1.0±0.2·10^{-2}$	0.977
	$NQO1_{dic}$	43.4±2.4	37.2±6.5	$2.8±1.4·10^{-4}$	0.934
129	$NQO1_{apo}$	62.1±2.3	32.6±2.3	$2.5±0.4·10^{-2}$	0.992
	$NQO1_{holo}$	76.5±1.2	18.8±1.2	$1.4±0.2·10^{-2}$	0.989
	$NQO1_{dic}$	36.6±5.8	36.2±6.9	$3.5±2.0·10^{-3}$	0.879
130	$NQO1_{apo}$	57.5±2.2	38.2±2.2	$2.3±0.3·10^{-2}$	0.994
	$NQO1_{holo}$	66.9±1.8	29.0±1.8	$1.8±0.3·10^{-2}$	0.991
	$NQO1_{dic}$	34.2±4.7	47.9±5.3	$5.7±1.7·10^{-3}$	0.957
131	$NQO1_{apo}$	46.8±2.4	49.4±2.4	$2.3±0.3·10^{-2}$	0.996
	$NQO1_{holo}$	53.5±2.6	42.6±2.6	$2.2±0.3·10^{-2}$	0.993
	$NQO1_{dic}$	29.2±2.2	60.7±2.3	$7.8±0.8·10^{-3}$	0.995
132	$NQO1_{apo}$	37.4±4.1	51.0±4.2	$1.5±0.3·10^{-2}$	0.982
	$NQO1_{holo}$	42.2±3.8	48.5±3.9	$1.1±0.3·10^{-2}$	0.979
	$NQO1_{dic}$	21.3±0.6	62.2±0.6	$8.9±0.3·10^{-3}$	0.999
133	$NQO1_{apo}$	18.0±2.2	48.0±4.5	$4.3±1.2·10^{-4}$	0.973
	$NQO1_{holo}$	11.6±1.1	61.4±1.7	$7.9±0.7·10^{-4}$	0.997
	$NQO1_{dic}$	10.5±0.6	17.2±0.8	$3.0±0.4·10^{-3}$	0.994
134-141	$NQO1_{apo}$	18.6±1.8	46.7±3.9	$3.7±0.9·10^{-4}$	0.980
	$NQO1_{holo}$	11.8±1.0	59.6±1.7	$7.0±0.6·10^{-4}$	0.997

	$NQO1_{dic}$	11.6±0.5	11.7±0.6	$2.9±0.5·10^{-3}$	0.991
142-145	$NQO1_{apo}$	5.1±0.2	2.5±0.3	$5.7±1.6 ·10^{-3}$	0.961
	$NQO1_{holo}$	3.6±0.2	3.1±0.2	$4.6±0.9 ·10^{-3}$	0.981
	$NQO1_{dic}$	2.6±0.3	2.7±0.4	$3.4±1.4 ·10^{-3}$	0.934
146-148	$NQO1_{apo}$	22.6±0.6	9.3±0.6	$3.6±0.7·10^{-3}$	0.982
	$NQO1_{holo}$	15.5±0.4	12.0±0.5	$4.1±0.4·10^{-3}$	0.993
	$NQO1_{dic}$	10.8±1.0	10.6±1.2	$2.8±1.0·10^{-3}$	0.952
149-155	$NQO1_{apo}$	56.9±0.6	19.1±0.6	$6.3±0.6·10^{-3}$	0.998
	$NQO1_{holo}$	44.8±1.3	27.4±1.5	$5.2±0.8·10^{-3}$	0.989
	$NQO1_{dic}$	31.5±3.1	31.5±3.7	$2.7±1.0·10^{-3}$	0.948
156	$NQO1_{apo}$	44.0±1.1	19.2±1.3	$3.6±0.7·10^{-3}$	0.982
	$NQO1_{holo}$	33.2±1.2	24.1±1.4	$3.9±0.7·10^{-3}$	0.987
	$NQO1_{dic}$	24.5±2.3	26.5±2.8	$2.3±0.8·10^{-3}$	0.959
157-164	$NQO1_{apo}$	17.3±0.6	21.7±1.0	$9.3±1.4·10^{-4}$	0.992
	$NQO1_{holo}$	6.6±1.1	23.5±1.6	$1.2±0.3·10^{-3}$	0.982
	$NQO1_{dic}$	5.9±0.7	20.5±1.0	$1.4±0.2·10^{-3}$	0.991
165	$NQO1_{apo}$	12.5±0.8	23.2±1.3	$7.4±1.3·10^{-4}$	0.989
	$NQO1_{holo}$	1.5±1.3	26.3±2.2	$6.7±1.8·10^{-4}$	0.974
	$NQO1_{dic}$	0.8±0.4	20.0±0.5	$1.2±0.1·10^{-3}$	0.998
166	$NQO1_{apo}$	SLNE			
	$NQO1_{holo}$	SLNE			
	$NQO1_{dic}$	SLNE			
167	$NQO1_{apo}$	SLNE			
	$NQO1_{holo}$	SLNE			
	$NQO1_{dic}$	SLNE			
168-173	$NQO1_{apo}$	SLNE			
	$NQO1_{holo}$	SLNE			
	$NQO1_{dic}$	SLNE			
174-175	$NQO1_{apo}$	SLNE			

	$NQO1_{holo}$	SLNE			
	$NQO1_{dic}$	SLNE			
176-177	$NQO1_{apo}$	SLNE			
	$NQO1_{holo}$	SLNE			
	$NQO1_{dic}$	SLNE			
178	$NQO1_{apo}$	SLNE			
	$NQO1_{holo}$	SLNE			
	$NQO1_{dic}$	SLNE			
179-181	$NQO1_{apo}$	SLNE			
	$NQO1_{holo}$	SLNE			
	$NQO1_{dic}$	SLNE			
182	$NQO1_{apo}$	0.6±0.4	8.8±0.5	$3.2±0.5 \cdot 10^{-3}$	0.990
	$NQO1_{holo}$	-0.3±0.2	9.1±0.2	$5.7±0.3 \cdot 10^{-3}$	0.999
	$NQO1_{dic}$	-0.5±0.2	9.1±0.2	$4.2±0.3 \cdot 10^{-3}$	0.998
183-185	$NQO1_{apo}$	1.4±0.6	15.2±0.7	$3.4±0.5 \cdot 10^{-3}$	0.992
	$NQO1_{holo}$	-0.4±0.2	16.0±0.3	$5.8±0.3 \cdot 10^{-3}$	0.999
	$NQO1_{dic}$	-0.7±0.3	16.0±0.3	$4.4±0.2 \cdot 10^{-3}$	0.999
186-189	$NQO1_{apo}$	1.9±0.7	21.7±0.9	$3.6±0.4 \cdot 10^{-3}$	0.994
	$NQO1_{holo}$	-0.5±0.3	22.9±0.4	$5.9±0.3 \cdot 10^{-3}$	0.999
	$NQO1_{dic}$	-0.9±0.7	22.9±0.5	$4.4±0.2 \cdot 10^{-3}$	0.998
190	$NQO1_{apo}$	13.9±0.9	21.4±1.1	$4.0±0.6 \cdot 10^{-3}$	0.991
	$NQO1_{holo}$	7.5±0.3	25.5±0.3	$5.4±0.2 \cdot 10^{-3}$	0.999
	$NQO1_{dic}$	7.5±0.2	24.9±0.2	$4.3±0.1 \cdot 10^{-3}$	0.999
191	$NQO1_{apo}$	42.4±1.9	20.6±2.1	$7.8±2.2 \cdot 10^{-3}$	0.965
	$NQO1_{holo}$	26.3±0.5	35.9±0.5	$4.5±0.2 \cdot 10^{-3}$	0.999
	$NQO1_{dic}$	27.0±0.8	34.5±0.9	$3.8±0.3 \cdot 10^{-3}$	0.997
192-201	$NQO1_{apo}$	63.5±5.1	25.7±5.1	$4.6±1.4 \cdot 10^{-2}$	0.955
	$NQO1_{holo}$	51.3±1.2	38.6±1.4	$3.9±0.4 \cdot 10^{-3}$	0.995
	$NQO1_{dic}$	52.1±1.0	37.9±1.2	$4.1±0.4 \cdot 10^{-3}$	0.997

202-203	$NQO1_{apo}$	32.4±3.0	60.3±3.4	$5.7±0.9 \cdot 10^{-3}$	0.988
	$NQO1_{holo}$	21.4±3.4	59.5±6.4	$5.2±1.7 \cdot 10^{-4}$	0.962
	$NQO1_{dic}$	22.4±3.9	63.5±7.1	$5.5±1.9 \cdot 10^{-4}$	0.958
204-205	$NQO1_{apo}$	5.3±3.3	53.5±4.0	$3.4±0.8 \cdot 10^{-3}$	0.979
	$NQO1_{holo}$	0.4±0.9	46.1±2.6	$2.5±0.4 \cdot 10^{-4}$	0.994
	$NQO1_{dic}$	1.0±1.2	49.7±3.1	$2.9±0.5 \cdot 10^{-4}$	0.991
206-211	$NQO1_{apo}$	-0.2±0.2	26.9±0.6	$2.2±0.1 \cdot 10^{-4}$	0.999
	$NQO1_{holo}$	-0.1±0.2	3.3±0.6	$2.3±1.0 \cdot 10^{-4}$	0.955
	$NQO1_{dic}$	-0.2±0.2	5.0±0.5	$3.4±1.0 \cdot 10^{-4}$	0.973
212	$NQO1_{apo}$	18.3±4.3	44.8±6.0	$1.2±0.6 \cdot 10^{-3}$	0.933
	$NQO1_{holo}$	16.0±4.8	43.6±5.8	$2.4±1.1 \cdot 10^{-3}$	0.935
	$NQO1_{dic}$	14.8±4.9	45.7±5.9	$2.5±1.1 \cdot 10^{-3}$	0.939
213-214	$NQO1_{apo}$	22.6±5.4	57.5±7.3	$1.4±0.6 \cdot 10^{-3}$	0.939
	$NQO1_{holo}$	21.2±5.7	60.5±7.4	$1.7±0.7 \cdot 10^{-3}$	0.944
	$NQO1_{dic}$	19.1±6.0	63±7.5	$1.9±0.8 \cdot 10^{-3}$	0.946
215-216	$NQO1_{apo}$	24.2±4.9	53.5±6.8	$1.3±0.6 \cdot 10^{-3}$	0.940
	$NQO1_{holo}$	23.2±5.1	56.6±6.8	$1.5±0.6 \cdot 10^{-3}$	0.946
	$NQO1_{dic}$	21.4±5.3	59.2±6.9	$1.7±0.7 \cdot 10^{-3}$	0.948
217-218	$NQO1_{apo}$	25.2±3.5	49.0±5.3	$9.6±3.4 \cdot 10^{-4}$	0.957
	$NQO1_{holo}$	24.7±3.6	52.2±5.2	$1.1±0.4 \cdot 10^{-3}$	0.962
	$NQO1_{dic}$	23.5±3.7	54.6±5.3	$1.1±0.4 \cdot 10^{-3}$	0.964
219	$NQO1_{apo}$	22.9±2.1	42.6±3.8	$5.6±1.6 \cdot 10^{-4}$	0.973
	$NQO1_{holo}$	22.2±2.0	44.6±3.6	$5.6±1.4 \cdot 10^{-4}$	0.978
	$NQO1_{dic}$	21.5±2.1	46.0±3.6	$6.3±1.6 \cdot 10^{-4}$	0.978
220-221	$NQO1_{apo}$	21.2±1.7	41.4±3.3	$4.9±1.2 \cdot 10^{-4}$	0.979
	$NQO1_{holo}$	20.1±4.5	43.5±2.9	$4.9±1.0 \cdot 10^{-4}$	0.985
	$NQO1_{dic}$	19.5±1.6	44.8±3.0	$5.4±1.1 \cdot 10^{-4}$	0.985
222	$NQO1_{apo}$	17.9±1.8	43.3±3.4	$5.4±1.3 \cdot 10^{-4}$	0.980
	$NQO1_{holo}$	14.7±1.7	45.6±3.2	$5.4±1.1 \cdot 10^{-4}$	0.984

	$NQO1_{dic}$	13.6±1.7	46.2±2.9	$6.1±1.2 \cdot 10^{-4}$	0.986
223-228	$NQO1_{apo}$	33.8±2.3	32.9±2.7	$3.8±0.9 \cdot 10^{-3}$	0.975
	$NQO1_{holo}$	25.9±1.8	39.0±2.2	$3.8±0.6 \cdot 10^{-3}$	0.989
	$NQO1_{dic}$	23.0±1.3	41.1±1.5	$3.4±0.4 \cdot 10^{-3}$	0.995
229-231	$NQO1_{apo}$	47.2±4.5	45.5±4.4	$5.7±0.8 \cdot 10^{-2}$	0.991
	$NQO1_{holo}$	49.7±5.6	42.5±5.5	$5.7±1.0 \cdot 10^{-2}$	0.984
	$NQO1_{dic}$	41.2±0.6	51.0±0.6	$1.7±0.4 \cdot 10^{-2}$	0.999
232	$NQO1_{apo}$	54.6±6.6	35.2±6.6	$3.0±1.1 \cdot 10^{-2}$	0.950
	$NQO1_{holo}$	62.5±4.2	28.8±4.2	$1.4±0.6 \cdot 10^{-2}$	0.943
	$NQO1_{dic}$	40.5±5.1	47.5±5.2	$1.2±0.4 \cdot 10^{-2}$	0.964
233-236	$NQO1_{apo}$	51.1±3.1	38.0±3.5	$6.6±1.7 \cdot 10^{-3}$	0.970
	$NQO1_{holo}$	49.8±2.8	41.4±3.1	$6.3±1.3 \cdot 10^{-3}$	0.979
	$NQO1_{dic}$	33.4±6.3	51.5±7.2	$4.8±1.9 \cdot 10^{-3}$	0.995
237	$NQO1_{apo}$	44.4±2.9	44.2±3.2	$5.8±1.2 \cdot 10^{-3}$	0.980
	$NQO1_{holo}$	42.2±2.6	48.6±2.9	$5.8±1.0 \cdot 10^{-3}$	0.987
	$NQO1_{dic}$	28.4±6.2	55.7±7.3	$3.6±1.4 \cdot 10^{-3}$	0.938
238	$NQO1_{apo}$	39.3±2.6	48.4±3.1	$3.7±0.7 \cdot 10^{-3}$	0.984
	$NQO1_{holo}$	36.2±2.6	55.2±3.0	$3.9±0.6 \cdot 10^{-3}$	0.989
	$NQO1_{dic}$	27.2±6.0	55.6±7.8	$1.7±0.8 \cdot 10^{-3}$	0.927
239-240	$NQO1_{apo}$	41.1±2.0	47.7±2.3	$6.2±0.8 \cdot 10^{-3}$	0.992
	$NQO1_{holo}$	34.7±1.7	55.6±1.9	$6.4±0.6 \cdot 10^{-3}$	0.996
	$NQO1_{dic}$	31.8±3.6	53.4±4.2	$5.0±1.0 \cdot 10^{-3}$	0.978
241	$NQO1_{apo}$	44.0±2.3	44.8±2.4	$1.0±0.2 \cdot 10^{-2}$	0.991
	$NQO1_{holo}$	38.7±1.4	50.5±1.4	$1.0±0.1 \cdot 10^{-2}$	0.997
	$NQO1_{dic}$	38.1±0.7	51.1±0.8	$8.4±0.4 \cdot 10^{-3}$	0.999
242	$NQO1_{apo}$	41.9±3.2	46.5±3.2	$1.6±0.3 \cdot 10^{-2}$	0.988
	$NQO1_{holo}$	37.5±2.8	51.0±2.8	$1.4±0.2 \cdot 10^{-2}$	0.992
	$NQO1_{dic}$	36.7±2.1	51.6±2.2	$1.2±0.1 \cdot 10^{-2}$	0.995
243-244	$NQO1_{apo}$	37.7±4.2	49.6±4.2	$2.0±0.4 \cdot 10^{-2}$	0.985

	$NQO1_{holo}$	34.1±3.6	53.7±3.6	$1.6±0.3 \cdot 10^{-2}$	0.989
	$NQO1_{dic}$	32.5±3.0	54.7±3.0	$1.5±0.2 \cdot 10^{-2}$	0.996
245-248	$NQO1_{apo}$	31.1±4.9	55.8±4.8	$2.8±0.5 \cdot 10^{-2}$	0.988
	$NQO1_{holo}$	26.7±4.9	59.5±4.8	$2.4±0.4 \cdot 10^{-2}$	0.988
	$NQO1_{dic}$	26.4±4.3	60.2±4.2	$2.1±0.4 \cdot 10^{-2}$	0.990
249-251	$NQO1_{apo}$	41.2±4.1	28.8±4.5	$6.4±2.8 \cdot 10^{-3}$	0.917
	$NQO1_{holo}$	34.5±4.8	34.4±5.4	$6.1±2.7 \cdot 10^{-3}$	0.916
	$NQO1_{dic}$	32.6±5.3	33.7±5.8	$7.7±3.7 \cdot 10^{-3}$	0.905
252-254	$NQO1_{apo}$	34.8±3.1	30.2±4.9	$8.5±4.5 \cdot 10^{-4}$	0.910
	$NQO1_{holo}$	27.7±4.4	35.7±6.7	$9.0±5.6 \cdot 10^{-4}$	0.882
	$NQO1_{dic}$	27.4±4.7	34.1±7.9	$6.8±5.1 \cdot 10^{-4}$	0.837
255	$NQO1_{apo}$	28.9±1.8	41.0±2.2	$2.4±0.4 \cdot 10^{-3}$	0.989
	$NQO1_{holo}$	22.5±1.8	48.3±2.4	$1.6±0.3 \cdot 10^{-3}$	0.990
	$NQO1_{dic}$	23.3±1.9	48.0±2.3	$2.6±0.4 \cdot 10^{-3}$	0.991
256-260	$NQO1_{apo}$	29.2±1.7	42.1±2.1	$2.4±0.4 \cdot 10^{-3}$	0.991
	$NQO1_{holo}$	21.9±1.9	50.3±2.5	$1.6±0.3 \cdot 10^{-3}$	0.990
	$NQO1_{dic}$	22.5±1.9	50.8±2.3	$2.7±0.4 \cdot 10^{-3}$	0.992
261	$NQO1_{apo}$	29.3±1.4	45.0±1.7	$2.7±0.3 \cdot 10^{-3}$	0.994
	$NQO1_{holo}$	21.6±1.7	52.2±2.2	$1.7±0.2 \cdot 10^{-3}$	0.993
	$NQO1_{dic}$	22.0±1.6	52.6±1.9	$3.0±0.3 \cdot 10^{-3}$	0.995
262	$NQO1_{apo}$	30.2±1.3	44.8±1.6	$2.7±0.3 \cdot 10^{-3}$	0.995
	$NQO1_{holo}$	21.9±1.6	51.9±2.1	$1.8±0.2 \cdot 10^{-3}$	0.993
	$NQO1_{dic}$	22.3±1.4	52.3±1.7	$3.1±0.3 \cdot 10^{-3}$	0.996
263	$NQO1_{apo}$	31.3±1.2	46.6±1.5	$2.9±0.3 \cdot 10^{-3}$	0.996
	$NQO1_{holo}$	23.5±1.6	53.3±2.0	$1.8±0.2 \cdot 10^{-3}$	0.993
	$NQO1_{dic}$	23.5±1.3	54.2±1.5	$3.2±0.3 \cdot 10^{-3}$	0.997
264-271	$NQO1_{apo}$	32.1±1.1	47.9±1.3	$3.1±0.3 \cdot 10^{-3}$	0.997
	$NQO1_{holo}$	23.8±1.5	55.3±1.9	$1.9±0.2 \cdot 10^{-3}$	0.995
	$NQO1_{dic}$	23.9±1.3	56.2±1.6	$3.4±0.3 \cdot 10^{-3}$	0.997

Table S2. Kinetic parameters for fitting of HDX kinetics to a three-parameter exponential function for 39 experimental peptides covering almost the entire NQO1 sequence. *SLNE* indicates *slow, little or no exchange*. *PF* indicates *poor fitting*.

Peptide	NQO1 protein	A_{burst} (%D)	A_{slow} (%D)	k_{slow} (s^{-1})	R^2
1-7	$NQO1_{apo}$	22.3±2.7	15.8±2.8	$1.3±0.6·10^{-2}$	0.914
	$NQO1_{holo}$	23.0±2.8	15.0±2.8	$1.2±0.6·10^{-2}$	0.898
	$NQO1_{dic}$	23.5±2.6	16.1±2.7	$1.0±0.5·10^{-2}$	0.912
7-10	$NQO1_{apo}$	SLNE			
	$NQO1_{holo}$	SLNE			
	$NQO1_{dic}$	SLNE			
11-19	$NQO1_{apo}$	69.1±1.7	17.4±1.7	$1.2±0.3·10^{-2}$	0.971
	$NQO1_{holo}$	32.8±0.8	37.4±1.1	$1.4±0.1·10^{-3}$	0.997
	$NQO1_{dic}$	31.2±0.2	6.0±0.2	$3.4±0.4 ·10^{-3}$	0.995
20-23	$NQO1_{apo}$	SLNE			
	$NQO1_{holo}$	SLNE			
	$NQO1_{dic}$	SLNE			
24-33	$NQO1_{apo}$	-1.3±0.6	19.5±0.9	$8.9±1.4 ·10^{-4}$	0.992
	$NQO1_{holo}$	-1.6±0.8	20.3±1.2	$9.0±1.8 ·10^{-4}$	0.987
	$NQO1_{dic}$	-1.5±0.6	19.8±0.9	$8.8±1.3 ·10^{-4}$	0.993
34-39	$NQO1_{apo}$	11.0±1.1	34.1±1.1	$1.4±0.1·10^{-2}$	0.997
	$NQO1_{holo}$	10.9±0.9	34.7±0.9	$1.4±0.1·10^{-2}$	0.998
	$NQO1_{dic}$	10.4±0.3	35.1±0.3	$1.3±0.1·10^{-2}$	1.000
42-45	$NQO1_{apo}$	1.6±4.2	77.8±6.7	$7.7±2.2 ·10^{-4}$	0.973
	$NQO1_{holo}$	0.6±2.6	74.4±4.7	$5.4±1.1 ·10^{-4}$	0.986
	$NQO1_{dic}$	2.0±2.3	72.4±4.2	$5.7±1.0 ·10^{-4}$	0.988
46-54	$NQO1_{apo}$	41.0±3.2	40.2±3.9	$2.3±0.7·10^{-3}$	0.964
	$NQO1_{holo}$	33.1±3.3	39.2±5.2	$7.9±3.4·10^{-4}$	0.939
	$NQO1_{dic}$	30.1±2.3	25.7±3.9	$6.5±3.1·10^{-4}$	0.923
55-59	$NQO1_{apo}$	83.6±2.6	7.1±2.6	$3.4±2.3·10^{-2}$	0.845

	$NQO1_{holo}$	58.2±0.7	32.9±0.7	$1.0±0.1·10^{-2}$	0.998
	$NQO1_{dic}$	58.4±0.5	32.1±0.7	$7.2±0.5·10^{-4}$	0.998
60-65	$NQO1_{apo}$	51.0±42.8	41.2±42.5	$1.2±1.0 ·10^{-1}$	0.892
	$NQO1_{holo}$	38.1±4.1	53.0±4.1	$2.8±0.5 ·10^{-2}$	0.991
	$NQO1_{dic}$	32.0±2.3	59.6±2.3	$1.4±0.2·10^{-2}$	0.996
66-73	$NQO1_{apo}$	52.3±5.3	36.6±5.2	$6.9±1.2·10^{-2}$	0.985
	$NQO1_{holo}$	26.9±1.5	63.5±1.5	$1.3±0.1·10^{-2}$	0.998
	$NQO1_{dic}$	26.3±3.8	59.4±6.2	$7.3±2.5·10^{-4}$	0.961
74-87	$NQO1_{apo}$	36.9±4.6	39.7±5.5	$3.3±1.4·10^{-3}$	0.931
	$NQO1_{holo}$	10.7±5.1	60.9±5.6	$7.0±1.8·10^{-3}$	0.970
	$NQO1_{dic}$	9.8±2.8	49.4±4.4	$8.1±2.4·10^{-4}$	0.971
88-92	$NQO1_{apo}$	SLNE			
	$NQO1_{holo}$	SLNE			
	$NQO1_{dic}$	SLNE			
92-96	$NQO1_{apo}$	22.5±4.1	33.2±9.6	$3.3±2.7·10^{-4}$	0.814
	$NQO1_{holo}$	22.9±4.1	30.6±9.9	$3.2±2.9·10^{-4}$	0.783
	$NQO1_{dic}$	24.5±4.0	29.6±9.0	$3.5±3.1·10^{-4}$	0.790
96-102	$NQO1_{apo}$	SLNE			
	$NQO1_{holo}$	SLNE			
	$NQO1_{dic}$	SLNE			
103-107	$NQO1_{apo}$	19.1±3.8	62.5±4.5	$4.1±0.8·10^{-3}$	0.981
	$NQO1_{holo}$	-6.8±2.3	35.5±2.8	$2.4±0.6·10^{-3}$	0.977
	$NQO1_{dic}$	SLNE			
108-113	$NQO1_{apo}$	1.9±4.8	44.9±5.7	$3.8±1.4 ·10^{-3}$	0.943
	$NQO1_{holo}$	-1.2±1.1	26.4±2.0	$5.9±1.4·10^{-4}$	0.981
	$NQO1_{dic}$	SLNE			
114-119	$NQO1_{apo}$	---	---	---	---
	$NQO1_{holo}$	---	---	---	---
	$NQO1_{dic}$	---	---	---	---

120-124	$NQO1_{apo}$	32.0±5.2	46.1±8.2	$8.4±4.9·10^{-4}$	0.893
	$NQO1_{holo}$	23.2±2.1	52.8±4.2	$4.4±1.0·10^{-4}$	0.980
	$NQO1_{dic}$	SLNE			
125-129	$NQO1_{apo}$	54.0±3.2	37.1±3.2	$4.5±0.6·10^{-2}$	0.991
	$NQO1_{holo}$	PF			
	$NQO1_{dic}$	PF			
129-132	$NQO1_{apo}$	36.6±2.9	62.3±2.9	$2.0±0.2·10^{-2}$	0.995
	$NQO1_{holo}$	40.9±2.6	58.9±2.6	$2.0±0.2·10^{-2}$	0.996
	$NQO1_{dic}$	19.7±0.5	77.7±0.5	$9.2±0.2·10^{-3}$	1.000
133-141	$NQO1_{apo}$	16.7±2.0	47.8±4.2	$3.9±1.0·10^{-4}$	0.977
	$NQO1_{holo}$	10.4±0.9	61.5±1.5	$8.0±0.6·10^{-4}$	0.998
	$NQO1_{dic}$	9.6±0.7	15.2±0.9	$2.6±0.5·10^{-3}$	0.987
142-145	$NQO1_{apo}$	SLNE			
	$NQO1_{holo}$	SLNE			
	$NQO1_{dic}$	SLNE			
146-156	$NQO1_{apo}$	47.8±1.2	20.6±1.4	$3.3±0.7·10^{-3}$	0.982
	$NQO1_{holo}$	33.2±0.9	25.7±1.1	$4.1±0.5·10^{-3}$	0.993
	$NQO1_{dic}$	23.3±2.1	22.8±2.6	$2.9±1.0·10^{-3}$	0.954
157-165	$NQO1_{apo}$	12.5±0.8	23.2±1.3	$7.4±1.3·10^{-4}$	0.989
	$NQO1_{holo}$	1.5±1.3	26.2±2.2	$6.7±1.8·10^{-4}$	0.975
	$NQO1_{dic}$	0.8±0.4	20.0±0.5	$1.2±0.1·10^{-3}$	0.998
166-173	$NQO1_{apo}$	SLNE			
	$NQO1_{holo}$	SLNE			
	$NQO1_{dic}$	SLNE			
174-181	$NQO1_{apo}$	SLNE			
	$NQO1_{holo}$	SLNE			
	$NQO1_{dic}$	SLNE			
182-189	$NQO1_{apo}$	-0.2±0.2	19.2±0.3	$2.9±0.1·10^{-3}$	0.999

	$NQO1_{holo}$	-1.1±0.4	19.8±0.5	$5.4±0.4·10^{-3}$	0.998
	$NQO1_{dic}$	-1.8±0.6	19.9±0.7	$4.1±0.4·10^{-3}$	0.996
190-201	$NQO1_{apo}$	74.6±1.9	16.1±2.1	$5.7±2.1·10^{-3}$	0.939
	$NQO1_{holo}$	49.8±1.3	35.4±1.5	$3.4±0.4·10^{-3}$	0.993
	$NQO1_{dic}$	51.2±1.7	31.1±2.0	$3.2±0.6·10^{-3}$	0.984
202-205	$NQO1_{apo}$	8.6±2.5	86.0±2.9	$4.8±0.5·10^{-3}$	0.996
	$NQO1_{holo}$	0.2±2.1	82.7±6.1	$2.7±0.5·10^{-4}$	0.989
	$NQO1_{dic}$	1.3±2.9	88.2±7.6	$2.9±0.7·10^{-4}$	0.982
206-211	$NQO1_{apo}$	-1.3±0.3	25.9±0.8	$2.4±0.2·10^{-4}$	0.998
	$NQO1_{holo}$	SLNE			
	$NQO1_{dic}$	SLNE			
212-218	$NQO1_{apo}$	17.2±5.5	63.0±7.3	$1.6±0.6·10^{-3}$	0.949
	$NQO1_{holo}$	17.1±5.9	66.7±7.6	$1.8±0.7·10^{-3}$	0.950
	$NQO1_{dic}$	15.6±6.1	69.5±7.8	$1.8±0.7·10^{-3}$	0.952
219-222	$NQO1_{apo}$	21.8±0.6	45.4±1.4	$3.0±0.3·10^{-4}$	0.998
	$NQO1_{holo}$	-1.0±1.0	49.3±3.1	$2.4±0.4·10^{-4}$	0.993
	$NQO1_{dic}$	-0.6±0.7	48.0±2.0	$2.8±0.3·10^{-4}$	0.996
223-228	$NQO1_{apo}$	37.1±1.5	30.2±1.6	$6.4±1.0·10^{-3}$	0.989
	$NQO1_{holo}$	29.9±1.2	34.9±1.4	$5.4±0.6·10^{-3}$	0.994
	$NQO1_{dic}$	26.6±0.8	37.6±0.9	$4.8±0.3·10^{-3}$	0.998
229-232	$NQO1_{apo}$	47.2±4.5	45.5±4.5	$5.7±0.8·10^{-2}$	0.991
	$NQO1_{holo}$	49.7±5.9	42.5±5.5	$5.7±1.0·10^{-2}$	0.983
	$NQO1_{dic}$	41.2±0.7	51.0±0.6	$1.7±0.1·10^{-2}$	1.000
233-238	$NQO1_{apo}$	35.9±3.1	50.1±3.7	$4.1±0.9·10^{-3}$	0.980
	$NQO1_{holo}$	35.9±3.2	54.5±3.8	$4.0±0.8·10^{-3}$	0.982
	$NQO1_{dic}$	20.8±7.7	58.0±9.3	$2.7±1.4·10^{-3}$	0.910
239-248	$NQO1_{apo}$	40.9±3.0	48.6±3.0	$1.4±0.2·10^{-2}$	0.989
	$NQO1_{holo}$	34.0±3.1	54.8±3.1	$1.5±0.2·10^{-2}$	0.991
	$NQO1_{dic}$	34.7±2.7	53.9±2.7	$1.2±0.2·10^{-2}$	0.992

249-254	$NQO1_{apo}$	34.8±3.1	30.2±4.9	$8.5±4.5·10^{-4}$	0.911
	$NQO1_{holo}$	27.7±4.4	35.7±6.7	$9.0±5.6·10^{-4}$	0.882
	$NQO1_{dic}$	27.4±4.7	34.1±7.9	$6.8±5.1·10^{-4}$	0.837
255-271	$NQO1_{apo}$	28.9±1.8	41.0±2.2	$2.4±0.4·10^{-3}$	0.989
	$NQO1_{holo}$	22.5±1.8	48.3±2.4	$1.6±0.3·10^{-3}$	0.990
	$NQO1_{dic}$	23.3±1.9	48.0±2.3	$2.6±0.4·10^{-3}$	0.991

Supplementary references

1. Medina-Carmona, E.; Neira, J.L.; Salido, E.; Fuchs, J.E.; Palomino-Morales, R.; Timson, D.J.; Pey, A.L. Site-to-site interdomain communication may mediate different loss-of-function mechanisms in a cancer-associated NQO1 polymorphism. *Scientific Reports* **2017**, *7*, 44352.
2. Kavan, D.; Man, P. MSTools-Web based application for visualization and presentation of HXMS data. *Int. J. Mass Spectrom.* **2011**, *302*, 53-58.
3. Faig, M.; Bianchet, M.A.; Talalay, P.; Chen, S.; Winski, S.; Ross, D.; Amzel, L.M. Structures of recombinant human and mouse NAD(P)H:quinone oxidoreductases: species comparison and structural changes with substrate binding and release. *Proc Natl Acad Sci U S A* **2000**, *97*, 3177-3182.
4. Asher, G.; Dym, O.; Tsvetkov, P.; Adler, J.; Shaul, Y. The crystal structure of NAD(P)H quinone oxidoreductase 1 in complex with its potent inhibitor dicoumarol. *Biochemistry* **2006**, *45*, 6372-6378.
